# Phylogenetic reconstruction in the Order Nymphaeales: ITS2 secondary structure analysis and *in silico *testing of maturase k (*matK*) as a potential marker for DNA bar coding

**DOI:** 10.1186/1471-2105-13-S17-S26

**Published:** 2012-12-07

**Authors:** Devendra Kumar Biswal, Manish Debnath, Shakti Kumar, Pramod Tandon

**Affiliations:** 1 Bioinformatics Centre, North Eastern Hill University, Shillong 793022, Meghalaya, India

## Abstract

**Background:**

The Nymphaeales (waterlilly and relatives) lineage has diverged as the second branch of basal angiosperms and comprises of two families: Cabombaceae and Nymphaceae. The classification of Nymphaeales and phylogeny within the flowering plants are quite intriguing as several systems (Thorne system, Dahlgren system, Cronquist system, Takhtajan system and APG III system (Angiosperm Phylogeny Group III system) have attempted to redefine the Nymphaeales taxonomy. There have been also fossil records consisting especially of seeds, pollen, stems, leaves and flowers as early as the lower Cretaceous. Here we present an *in silico *study of the order Nymphaeales taking maturaseK (*matK*) and internal transcribed spacer (ITS2) as biomarkers for phylogeny reconstruction (using character-based methods and Bayesian approach) and identification of motifs for DNA barcoding.

**Results:**

The Maximum Likelihood (ML) and Bayesian approach yielded congruent fully resolved and well-supported trees using a concatenated (ITS2+ matK) supermatrix aligned dataset. The taxon sampling corroborates the monophyly of Cabombaceae. *Nuphar *emerges as a monophyletic clade in the family Nymphaeaceae while there are slight discrepancies in the monophyletic nature of the genera *Nymphaea *owing to *Victoria-Euryale *and *Ondinea *grouping in the same node of Nymphaeaceae. ITS2 secondary structures alignment corroborate the primary sequence analysis. Hydatellaceae emerged as a sister clade to Nymphaeaceae and had a basal lineage amongst the water lilly clades. Species from *Cycas *and *Ginkgo *were taken as outgroups and were rooted in the overall tree topology from various methods.

**Conclusions:**

*MatK *genes are fast evolving highly variant regions of plant chloroplast DNA that can serve as potential biomarkers for DNA barcoding and also in generating primers for angiosperms with identification of unique motif regions. We have reported unique genus specific motif regions in the Order Nymphaeles from *matK *dataset which can be further validated for barcoding and designing of PCR primers. Our analysis using a novel approach of sequence-structure alignment and phylogenetic reconstruction using molecular morphometrics congrue with the current placement of Hydatellaceae within the early-divergent angiosperm order Nymphaeales. The results underscore the fact that more diverse genera, if not fully resolved to be monophyletic, should be represented by all major lineages.

## Background

The Basal angiosperm Order Nymphaeales is a group of water-living flowering plants. Though the group is taxonomically small, it has great significance in understanding the early evolutionary pattern of angiosperms. Classification of this Order varies from recognition of two to four families. A lot of progress has been made in recent years in understanding both the taxonomic position of Nymphaeales in the angiosperm tree and the relationship within the water lily clade [1-3].

Usually, two families, Cabombaceae and Nymphaeaceae are recognised. The Cabombaceae comprises the genera *Cabomba *and *Brasenia *and Nymphaeaceae comprise six genera: *Euryale*, *Ondinea*, *Victoria*, *Barclaya*, *Nuphar *and *Nymphaea*, the largest and most cosmopolitan in nature. Until recently, Hydatellaceae was placed among the monocots in previous systems and was placed with Poales, but a recent study with multi-marker plastid dataset found that the family belongs to Nymphaeales and includes two genera (*Hydatella *and *Trithuria*), which is restricted to Australiasia and India [4].

The Order Nymphaeales was considered to include the genera *Nelumbo *and *Ceratophyllum *as per earlier taxonomic treatments based on morphology [5-8]. However, in recent times with the use of modern molecular bio-markers, *Nelumbo *and *Ceratophyllum *are excluded, thereby, substantiating the monophyly of Nymphaeles [8-10]. This provided an impetus for revaluation of morphological characters that revealed the presence of certain features such as tricolpate pollen or epicuticular wax tubules in *Nelumbo *thereby further substantiating its exclusion from Nymphaeales [5,11].

Hydatellaceae as it represents the single exception in an otherwise relatively harmonious congruence between the traditional and molecular circumscription of the monocot clade, the structural diversity of this remarkable family is of considerable interest. They are small and inconspicuous plants that received little attention from botanists prior to their taxonomic reassignment to the basal angiosperms. It would be really interesting to review our current knowledge on this species-poor but interesting family that has only recently been discovered in India [4].

Morphological and molecular data generally indicate a close association of *Cabomba *and *Brasenia *thereby affirming the monophyly of the family Cabombaceae [12,13], whereas the monophyly of the family Nymphaeaceae is yet to get much support from the taxonomic community.

DNA barcoding has become an indispensable tool in identifying biological specimens using a short standardized region of both genomic as well as extra-chromosomal DNA very much in the way what universal product codes do for identification of consumer goods. Research community interested in DNA barcodes want to place their query sequences within the taxonomic hierarchy which is achieved by conventional sequence similarity search methods viz., Basic Local Alignment Search Tool (BLAST), Fast Alignment (FASTA) etc. that are often twitched to overcome biological mutations or sampling bias and this, in turn, poses tricky issues like successful tracking of minuscule sequence variations observed among closely related species. A step further, character based similarity relying on common ancestry is also employed in the form of phylogenetic trees or in the form of implicit hierarchic taxonomic descriptors [14]. These methods heavily depend on multiple sequence alignments (MSA) which in fact, is a challenge as the barcoding requirements are contradictory to the very objective of MSA, i.e., looking for hyper variable regions to delineate the closely related species and yet be highly conserved for allowing design of universal PCR primers. Keeping these in mind, selecting a core barcode abiding the three important barcoding principles (standardization, minimalism and scalability) still remains a challenge for plant DNA barcoding unlike animal DNA barcoding. The standard animal Cytochrome oxidase (COI) DNA barcode being a haploid and uniparentally inherited with a single locus exhibiting high levels of discriminatory power fits well into the above barcoding criteria [15].

COI is a protein coding marker with high copy numbers per cell devoid of microinversions (frequent mononucleotide repeats) and drastic length variation with developed primer sets that aid in routine recovery of high quality sequence from animal clades and sequence recovery from poorly preserved samples as well [15]. Finding a standard plant barcode analogue to COI in animals has proved difficult and COI from plant mitochondrial DNA (mtDNA) generally exhibits low nucleotide substitution rates thereby making it unsuccessful for universal plant barcoding initiatives. There are core research groups who have worked both *in silico *and *in vitro *suggesting multiple plastid markers but eventually couldn't arrive at a conclusion [15] and thus maturase K (matK) still holds good as a suitable substitute plant barcode that can be considered the animal barcode COI analogue [16].

matK is one of the most rapidly evolving coding regions in the plastid genome but unfortunately poses difficulty in PCR amplification with already existing universal primer sets especially in non-angiosperms contrary to another barcode region ribulose-bisphosphate carboxylase (rbcl) gene which is easy to amplify, sequence and align despite having modest discriminatory power [17]. Hence, two marker plastid barcodes (rbcl+matK) are suggested as core barcodes until further works on matK universal primer development are a success. With these two joint challenges (matk primers in want of improvement and uncertainty in discriminatory powers of two plastid marker (rbcl+matk) system), continued sequencing and exploration of new possibilities in non-coding markers viz. trnH-psbA and internal transcribed spacers (ITS1 & ITS2) are harnessed to formalize the routine incorporation of other potential non-coding markers into plant barcoding design systems [17].

Officially rbcl+matK combination has been approved by Consortium for the Barcode of Life (CBOL) as a global DNA barcode for land plants while trnH-psbA are still under scrutiny as a backup barcoding locus. There have been studies for ferns with matK+rbcl and trnH+psbA loci with the former providing high discriminatory power, supporting their use as the official DNA barcode [17]. Another research study has validated use of ITS2 as novel DNA barcode for medicinal plant identification as ITS2 sequences are considered potential phylogenetic markers at genus and species levels. Six parameters viz. average interspecific distance (K2P) between all species in each genus, average theta prime (θ'), where θ' is the mean pair wise distance within each genus with more than one species, smallest interspecific distance i.e., the minimum interspecific genetic distance within each genus with at least two species, average intra specific divergence (K2P difference), theta (θ) where θ is the mean pairwise distance within each species with at least two representatives and average coalescent depth (i.e., maximum intra-specific distance within each species with at least two representatives) were determined taking several plastid and ribosomal intergenic marker regions where ITS2 scored high exhibiting highest level of variation with all the parameters thereby accounting for ITS2 as a suitable marker with authentication ability [18].

Looking into these intriguing questions about phylogenetic relationships in Nymphaeales, we designed an *in silico *study using *matK *and ITS2 sequences available on the public domain covering all genera of Nymphaeales. Till date there are no reports on plant DNA barcoding approach where both matK and ITS2 are taken together and phylogenetic studies made. In case of water lilies, molecular identification and barcodes have been reported only for the genus *Nymphaea *and that too taking sequences from the rpoC1 gene and trnH-psbA spacer regions (which are still under assessment as backup loci by CBOL) and use of inter-simple sequence repeat (ISSR) for species identification and differentiation of *Nymphaea *cultivars and natural populations [19]. The present study aims at using both matK and ITS2 as markers for elucidating the plant species of the order Nymphaeales using combined fusion matrix of both the markers, capturing the phylogenetic signals through molecular morphometrics for the ITS2 region and finding novel motifs that can be tested as PCR primers in design of potential barcodes at genus level for rapid and accurate plant identification across the three different families of Nymphaeaceae, Cabombaceae and Hydatellaceae without morphological characters.

ITS2 has common core secondary structures across eukaryotes that serve as a double-edged tool. The ITS2 region of the nuclear rDNA cistrons is widely used for phylogenetic analyses at the genus and species levels and also at the higher taxonomic ranks using comparisons of primary sequence. Although potential transcript secondary structure homology is often utilized to aid alignment in comparisons of ribosomal gene sequences, such consideration has rarely been applied to ITS primarily because secondary structures for its transcript were not available. Hence, the value of applying ITS2 RNA transcript secondary structure information to improve alignments, that in turn, allows comparisons at even deeper taxonomic levels harnessing the evolutionarily conserved subportions of ITS2 has become apparently necessary for positioning of the multimolecular transcript processing machinery amongst eukaryotes and thus makes ITS2 a valuable tool both for primary sequence analysis and molecular morphometrics [20].

Although individualistic approach for different barcodes exist in addressing several issues in plant barcode designing, there is a general need for integrating a range of analytical routine into a common work flow to provide comparable informatics support for existing molecular data on the public domain.

For the present *in silico *study three principal objectives have been envisaged:

1. Phylogenetic reconstruction of Nymphaeales based on *matK *genes and ITS2 using a combined approach of gene trees and species trees (using super matrix of concatenated loci) and testing monophyly of the genus *Nymphaea*.

2. Evaluation of the phylogenetic utility of *matK *as a potential marker for motif hunting and DNA barcoding.

3. ITS2 secondary structure prediction in the order Nymphaeales and alignment of secondary structures to produce a consensus Nymphaeale phylogeny.

## Results

### Sequence analysis and phylogeny reconstructions

The sequences of ITS2 and matk were aligned separately with clustalW program [21] and manually edited and the resultant aligned files were concatenated using FASconCAT version 1.0 [22] (Additional File 1). For the insertion of gaps, attention was given to both the potentially inserted sequence and its neighbouring sequences. A gap was inserted only when it prevented the inclusion of more than two substitutions among closely adjacent nucleotides in the alignment. For the placement of gaps, the recognition of sequence motifs was given priority as per alignment rules for length-variable DNA sequences [23]. Giving priority to a motif can result in insertions that are correctly aligned as non-homologous (i.e. with different positional extensions) although sequence similarity would warrant their inaccurate placement under the same column [24]. Individual positions in homo-nucleotide strings of different lengths (poly-As or -Ts) are considered to be of uncertain homology [25] and are therefore excluded. Slipped strand mispairing [26] is likely to have led to numerous length mutational events involving one to several nucleotides. As only nucleotides of the same kind are involved, accurate motif recognition is not possible. Entire indels of the same positional extension and of complete sequence similarity were very easily assessed as primary homologues and consequently placed in the same column(s) of the alignment. During primary homology assessment, no inference had to be made regardless of whether the length mutational event occurred in a common ancestor of all taxa sharing it or in parallel in different lineages. This is analogous to the fact that the synapomorphic status of a substitution in a particular position is not inferred in the alignment process. Recognition of a repeat motif was regarded as further evidence for correctly recognizing a length mutational event. The final concatenated supermatrix included 51 taxa with 1875 characters.

### Maximum Likelihood (ML) analyses

**Figure 1:**
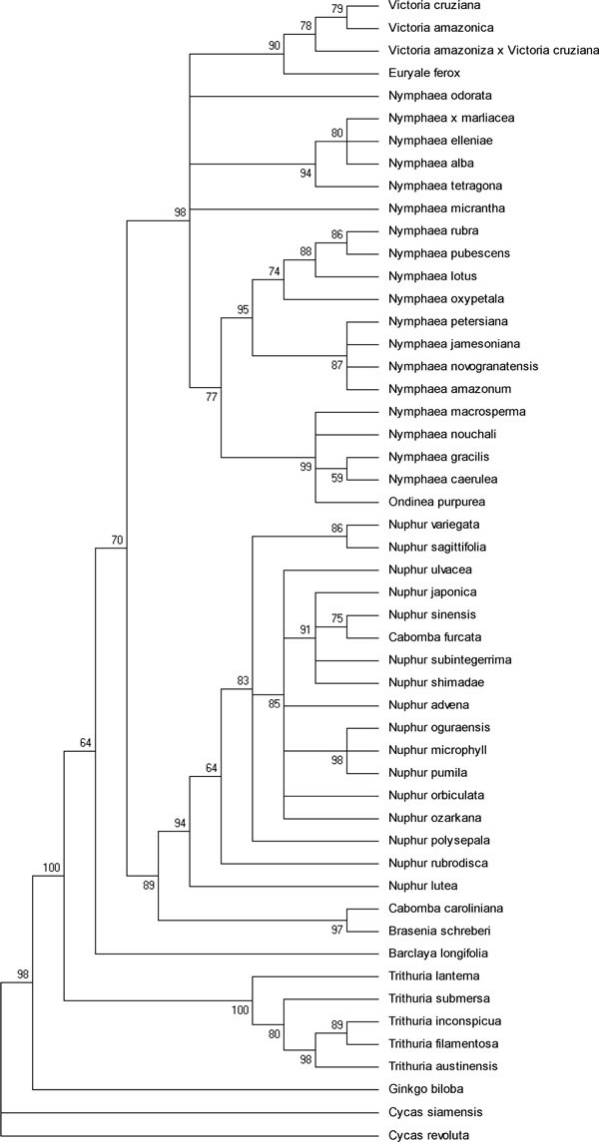
**ML topology of Nymphaeales from the aligned concatenated super matrix dataset using PhyML 3.0**. Phylogeny reconstruction of the Order Nymphaeales based on concatenated dataset of two different loci (ITS2+ matK) using a taxon set of 51 taxa (including three outgroup taxa, *Cycas revoluta*, *Cycas siamensis *and *Ginkgo biloba*) with aLRT values (best ML tree, majority rule, aLRT values similar to 100 bootstrap replicates)

Phylogenetic tree analysis was carried out using PhyML 3.0 [27] with approximate likelihood ratio test (aLRT) which is much faster than bootstarp and is close to Bayesian posteriors. We then implemented a Shimodaira-Hasegawa-like procedure [28], which is non-parametric and resembles well with bootstrap outcomes. The default substitution model HKY85 with gamma shape parameter of 2.716 and transition/transversion ratio of 3.064 was considered for computing the ML tree (Figure 1) that showed several groupings of the family Nymphaeaceae and contributed to the monophyly of the different genera therein viz., *Euryale, Barclaya, Nuphar*, *Nymphaea*, and *Victoria *except *Ondinea *that grouped with *Nymphaea*. There were slight variations in the placing of some species of Nymphaeaceae especially from the genus *Nymphaea *that clustered with other groups thereby accounting to its genetic variability. *Ginkgo *and *Cycas *representatives were taken as outgroup and were rooted in the overall tree topology with strong bootstrap values. Primarily three clades were resolved: Cabombaceae (with the genera *Cabomba *and *Brasenia*), Nymphaeaceae and Hydatellaceae (with the genera *Trithuria*). The grouping of *Trithuria *sps and placing of the family in the basal grade close to the outgroups reflected Hydatellaceae and Nympheaceae to be sister groups and that Hydatellaceae belonged to a more primitive basal angiosperm lineage.

### Bayesian analyses and split networks

**Figure 2:**
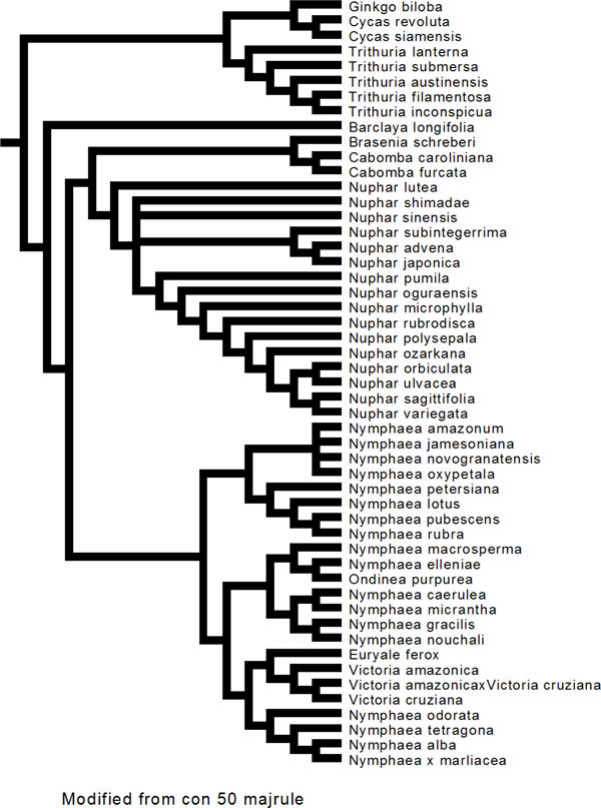
**Bayesian Phylogram (majority rule consensus tree) inferred from the aligned supermatrix dataset (ITS2 + matk)**. Nymphaeale phylogeny reconstruction using sequence evolution model using GTR with 10 million generations, sample frequency, 1000, burn-in: 10% discarded in MrBayes 3.2. The third family Hydatellaceae represented by the genus *Trithuria *sps formed a sister basal lineage to Cabombaceae and Nymphaeaceae and clustered with the outgroup (*Cycas *and *Ginkgo*).

**Figure 3:**
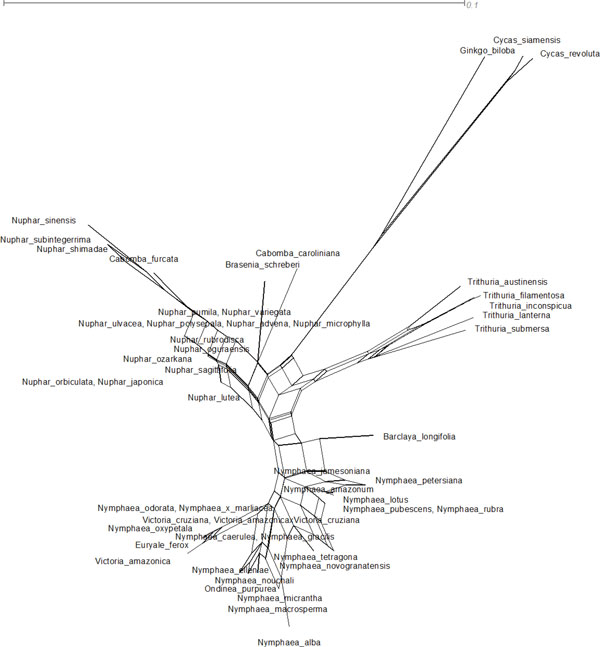
**Median-joining network graphs with uncorrected p distances inferred with Splitstree version 4.10 from the supermatrix (ITS2+ matK)**.

The supermatrix dataset of ITS2 and matK was exported in nexus format for MrBayes [29] in the Mesquite program V2.75 [30]. Bayesian analysis retained the same topology and supported the branches with a consensus 50 majority rule (Figure 2) though the basal lineage to the Nympheales group were represented by both the Hydatellaceae and *Cycas, ginkgo *outgroup. Our analyses showed that an exclusion of randomised sections improved the resolution between the different genera of the family Nymphaeaceae. The monophyly of the order Nympheales has been favoured by earlier studies [4-6]. Therefore, we conclude that more genes are necessary to robustly resolve Nymphaeale clade as well as relationships between Nymphaeaceae and Hydatellaceae. The above observations on the genetic variability in the family Nymphaeaceae prompted us for a median-joining and network analysis, which was performed using SplitsTree4 [31] with the variable positions in the aligned concatenated ITS2 and *matK *data. The median network tree exhibited primarily three groups accounting for the monophyly of *Nymphaeaceae, Cabombaceae and Hydatellaceae with Cycas and Ginkgo *as outgroups (Figure 3). Though the network analysis strongly corroborated the results of the MrBayes and ML analyses (Figures 1 and 2), Neighbor Network graphs give an indication of noise, signal-like patterns and conflicts within a super matrix aligned dataset.

### Molecular clock rates, dS/dN analysis

**Figure 4:**
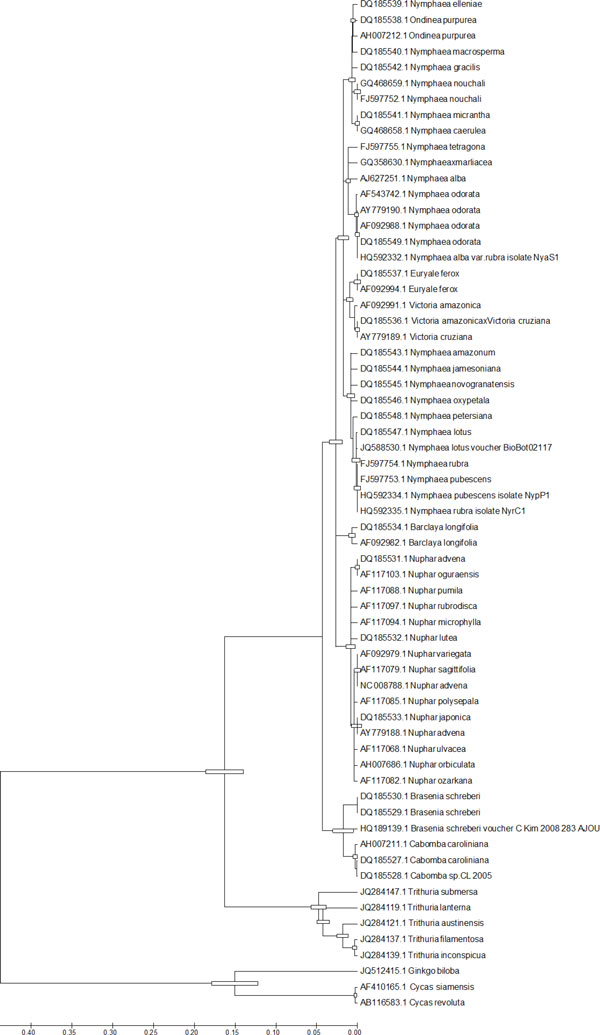
**Maximum Parsimonious tree of Nymphaeales using molecular clock test of matK sequence**. Molecular clock test performed by comparing the ML value for the given topology with and without the molecular clock constraints under Jukes-Cantor (1969) model (+G). Differences in evolutionary rates among sites modeled using a discrete Gamma (G) distribution. The null hypothesis of equal evolutionary rate throughout the tree was rejected at a 5% significance level (P < 1.20575200719741E-58). The analysis involved 64 nucleotide sequences. Evolutionary analyses were conducted in MEGA 5.

The molecular clock based on the molecular clock hypothesis (MCH)) is a technique in molecular evolution that uses fossil constraints and rates of molecular change to deduce the time in geologic history when two species or other taxa diverged and estimates the time of occurrence of events called speciation or radiation. Likelihood ratio test of the molecular clock where the ML value for a given tree assuming the rate uniformity among lineages is compared. The test rejects the null hypothesis when applied to data sets containing many sequences or long sequences as the strict equality of evolutionary rates among lineages is frequently violated. Conversely, the estimates of branch lengths, and thus interior node depths, in a tree obtained under the assumption of a molecular clock can be useful to generate a rough idea about the relative timing of sequence divergence events [24]. Comparing the ML value in Jukes-Cantor model [32] performed the molecular clock test and a Maximum Parsimony (MP) tree (Figure 4) was generated for the *matK *dataset. The molecular clock test output is outlined in Table 1.

**Table 1:** Results from a test of molecular clocks using the Maximum Likelihood method of Nymphaeales matK sequence.

	lnL	(+G)	(+I)
With Clock	-4525.281640.931	n/a	n/a
Without Clock	-4304.5181262.42	n/a	n/a

The codon-based z-test was carried out by setting the model to Syn-Nonsynonymous and Nei-gojobori test. The resulting matrix displayed (dS-dN) values above the diagonal and p values below the diagonal (Additional Files 2 &3). The test was carried out for both the hypotheses of positive selection and purifying selection. When (dS-dN) value is positive it exhibits purifying selection and to test that in reality p value less than 0.05 supports significant purifying selection. Selecting the p-value of 1.0 and then looking for the corresponding (dS-dN) value exhibited positive values thus rejecting the hypothesis of positive selection as dS > dN, i.e., silent mutations or purifying selection outnumbered non-synonymous mutations. Hence, we can conclude that the evolution of *matK *genes has been under strong purifying selection, suggesting their role in the evolution of Nymphaeales.

### Motif identification and matching

A total of 27 unique *matK *motifs are identified by the MEME software [33] and subsequently validated by the MAST tool [34]. We have reported three motifs each for the genera *Brasenia, cabomba, Barclaya, Euryale, Nuphur, Nymphaea, Ondinea*, *Victoria *and *Trithuria *along with their E-value, p-value [35] and similarity among themselves as outlined in (Additional File 4). In the proposed motif analysis that can be further tested for designing barcodes the same sets of sequences were used both to generate databases and as query sequences for both BLAST [36] and MAST. BLAST queries were run without filtering. Before generating the database with MAST the sequences were run through a PERL script that added a reverse complement for each sequence in order to ensure that query sequences would match the database in either the forward or the reverse orientation.

### ITS2 secondary structure and analysis: a double edged tool

**Figure 5:**
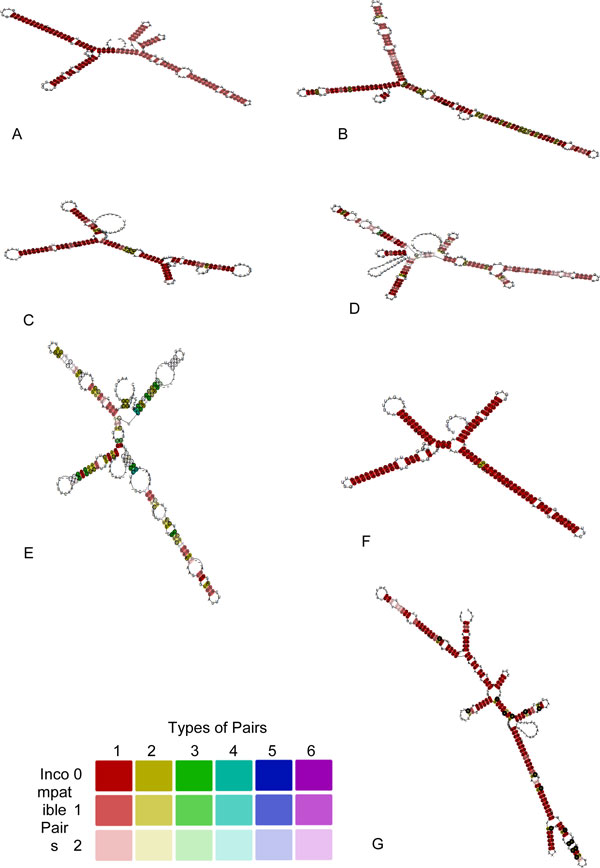
**ITS2 Consensus secondary structures of Nymphaeales with color legend using RNAz and LocaRNA**. Validation of conserved ITS2 secondary structures across the three Nymphaeale families (Cabombaceae, Nymphaeaceae and Hydatellaceae). The three families are represented by the genera A. *Brasenia*, B. *Cabomba*, C. *Euryale*, D. *Nuphur*, E. *Nymphaea *F. *Victoria *G. *Trithuria*. Standard nucleotide ambiguity codes are used.

In the present study representative sequences from ITS2 (Additional File 5 &9) were analyzed in RNAz [37] secondary structure alignment web server program with default parameters to assess the overall secondary structure analysis that were carried out through several computational approaches. The ITS2 dataset was first aligned in clustalW [21] and then subjected to RNA structure folding genus wise in the three families (Nymphaeaceae, Cabombaceae and Hydatellaceae). As can be followed from the figures arrow pointing to the right indicates forward reading direction related to the uploaded alignment (Figure 5, Additional File 6). In alignments with P > 0.5 the functional RNA is predicted. The higher this value, the more confident is the prediction. In standard analysis mode the results are outlined in several windows probability value s both forward and reverse reading frames. Here we have taken the results of those window predictions that have a high probability value among all the predicted window outputs. The location, length, number of sequences in the alignment, reading direction, consensus minimum free energy (MFE) structure values, mean z-score etc. are given in a tabular format for each group along with their consensus alignment and structures (Figure 5, Additional File 6). The consensus MFE is the average folding energy from the standard energy model. The second term of the consensus MFE i.e. covariance contribution indicates "bonus" or "penalty" energies for compensatory/consistent and inconsistent mutations, respectively. 'Combinations/Pair' is a value that helps quantifying compensatory/consistent mutations. It is the number of different base pair combinations in the consensus structure divided by the overall number of pairs in the consensus structure. Z-score was calculated by RNAz. A z-score is calculated as z = (m-μ)/σ, where μ and σ are the mean and standard deviations, respectively, of the MFEs with comparable random samples. Negative z-scores indicate that a sequence is more stable than expected by chance. All the representative structures spanning the family of Nymphaeaceae and Cabombabceae show negative values thereby indicating stable secondary structures (Figure 5, Additional File 6). To further validate the conservedness of ITS2 regions in the Order Nymphaeales we subjected the ITS2 dataset to LocaRNA [38] prediction tool that simply takes raw sequences rather than an aligned file. LocaRNA itself computed for global consensus regions and gave an alignment file along with the common core secondary structures across different genera in the order Nymphaeales (Additional File 7). Compatible base pairs are colored, where the hue shows the number of different types C-G, G-C, A-U, U-A, G-U or U-G of compatible base pairs in the corresponding columns that reflects sequence conservation of the base pair. The saturation decreases with the number of incompatible base pairs and hence, indicates the structural conservation of the base pair. All the consensus structures clearly exhibit the monophyletic nature at the genus level in both the families of Nymphaeales.

### Primary sequence-secondary structure alignment

**Figure 6:**
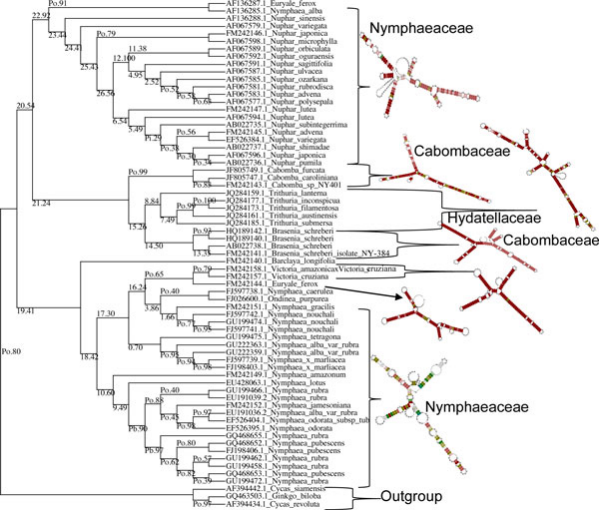
**Profile Neigbour Joining (PNJ) tree from primary sequence- secondary structure alignment of Nymphaeale ITS2 data using 4SALE and ProfDistS**. Simple correction Jukes and Cantor formula (Jukes and Cantor, 1969) operated on sequence-structure alignments. Based on the GTR RNA sequence-structure specific substitution model evolutionary distances between sequence-structure pairs are estimated by maximum likelihood and are also extended on the profile level. The group Hydatellaceae clustered with Cabombaceae and emerged as a sister clade to Nymphaeaceae. Consensus bootstrap values with 100 replicates are shown next to branches and ProfDistS output tree file viewed in NJplot. Tree viewing Profiles are marked by "Pi" (profile generated by identity threshold), "Pb" (profiles generated by bootstrap threshold) and "Po" (old profile generated in a previous iteration).

To further extend our analysis and compare the multi gene supermatrix dataset species tree with ITS2 secondary analysis of the species in the order Nymphaeales, we carried out sequence-structure alignment using 4SALE 1.7 [39] and (Profile-) Distance based phylogeny on sequence-structure alignments (ProfDistS) [40] and NJplot [41]. The tree reconstructing algorithm operated on a 12 letter alphabet comprised of the four nucleotides in three structural states (unpaired, paired left, paired right, e.g. 'A.', 'A(', 'A)', 'U.', etc.) and combined a general time reversible (GTR) model [42] on the sequence level with a substitution model on morphological features of the structures. Based on the GTR RNA sequence-structure specific substitution model [39] evolutionary distances between sequence-structure pairs were estimated by maximum likelihood and are also extended on the profile level. The secondary structure alignment tree (Figure 6) was then achieved on the RNA sequence-structure level with the help of the pipeline consisting of the ITS2 database, the sequence structure alignment editor 4SALE [39] and the phylogentic reconstruction tool ProfDistS [40]. The secondary structure alignment tree could resolve the monophyletic nature of the three families Nymphaeaceae, Hydatellaceae and Cabombaceae within the order Nymphaeales with supportive bootstrap values (Figure 6). *Cycas *and *Gingko *were rooted as outgroups. The members of the Hydatellaceae family clustered together with the members of Cabombaceae and this indicates Hydatellaceae to be a part of a larger ancient lineage with more evolved and diverse modifications for aquatic life habitat than previously recognized. The overall tree topology congrued with the earlier results of ML, Network analysis and Bayesian phylogeny. Further mountain graphs for RNA secondary structure diagrams for ITS2 were computed in MATLAB R2012a environment. Each base is represented by a dot in a two-dimensional plot, where the base position is in the abscissa (x-axis) and the number of base pairs enclosing a given base is in the ordinate (y-axis). The mountain peaks with blue dots (paired) and red dots (unpaired) are plotted across the Nympheales taking 3 representative sequences from each genus (Additional File 8) and the results were in agreement with that of LocaRNA results.

## Discussion

Populations are relatively isolated from one another where species dispersal is poor thereby resulting in slow individual neutral mutational variants spreading throughout a species range and thus for a species to attain monophyly for a particular loci it will be comparatively slower than species whose populations are connected with a regular gene flow. Hence, species-specific barcodes are literally difficult with poorly dispersed species. Since plastid markers in water lilies are paternally inherited, and travel in pollen, they potentially cover larger distances and have a better resolution power at species level delineation exhibiting consistently greater congruence with morphological species boundaries than maternally inherited mitochondrial markers [15]. There are also instances where multiple species are reported to share plastid DNA haplotypes yet remain distinct for nuclear markers like nrITS which is again explained by their dispersal ability i.e., plastid DNA is poorly dispersed compared to nrITS and thus a combined approach of marker selection with varied dispersal ability provide an optimal choice of augmenting plant barcodes with nuclear markers [18].

The markers used in our study are from both plastid and nrDNA with matK and ITS2 combination that were subjected to multiple sequence alignment and refined with Mesquite. Postulated indels were treated as missing data and prealigned marker datasets were concatenated to produce a fusion matrix and a supertree was generated. The combo approach of ITS2+matK had the combined effect of idiosyncratic behaviour of both the markers that potentially contributed to species grouping across different clades of the order Nymphaeales.

This study represents the exclusive molecular dataset for *matK *genes as potential markers for motif discovery till date. Due to a relatively high percentage of variable and informative characters, our dataset not only comprises a high number of informative characters for Nymphaeales but also characterised by low degrees of homoplasy and a strong phylogenetic signal. The ML method as well as the bayesian approach yielded the same results with an exactly matching topology and well supported nodes. The results confirm several earlier hypotheses on phylogenetic relationships of the Order Nymphaeales and corroborate the monophyly of Nymphaeaceae and Cabombaceae, which has been convincingly mentioned before based on integrated morphological, anatomical and molecular characters [43]. *Barclaya *serves as an outgroup to the monophyletic grouping of *Nymphaea*, *Ondinea*, *Victoria *and *Euryale*. It also supports the *Victoria-Euryale *grouping that was long predicted based on seed morphology and presence of spines [43]. Though Nymphaeales is a monophyletic group within the basal angiosperms, the monophyly of Nymphaeaceae is not fully convincing owing to *Victoria-Euryale *and *Ondinea *grouping. The classification of *Nymphaea *in India has been reported to be confusing, molecular taxonomic revision of four Indian representatives of the genus namely *N. nouchali, N. pubescens, N. rubra *and *N. tetragona *based on ITS, trnK intron and *matK *gene have been carried out by us earlier. Molecular evidence was in disagreement about the taxonomic identity of one specimen of *N. nouchali *and indicated a probable misidentification of *N. tetragona*. Interestingly, sequence analysis had revealed lack of or low sequence divergence between *N. pubescens *and *N. rubra *[44-46]. Further in the present study we tried to track down evolutionary relationships among the genera of the order Nymphaeales by comparing the nucleotide sequences of the plant genomic and chloroplast DNA. For the first time, we have banked upon a large dataset from publicly available *matK *and ITS2 markers for discussing Nymphaeale phylogeny with a molecular morphometrics approach. Several authors [48,49] considered assigning *Barclaya *to a separate family, Barclayaceae as they were of the argument that the genus *Barclaya *is quite distinct in terms of its palinological features, the structure of the ovule and the karyotype and in the present study our secondary structure alignment data (Figure 6) indicate that the region analysed in these studies is too short to enable verification of a phylogenetic hypothesis though we have got favourable results for considering *Barclaya *to be placed in a different family and with more diverse dataset we can target appropriate phylogenetic signals for considering *Barclaya *in a separate family.

The fact that *Nymphaea *and *Victoria *are the sister genera in our study is quite expected as both are highly evolved representatives of Nymphaeales. The recent study on Hydatellaceae [4] that identified it as a new branch near the angiosperm basal phylogeny was also reflected in our molecular morphometric analysis. Earlier ideas on the relationships of Hydatellaceae with the monocot family Centrolepidaceae and their current placement within the early-divergent angiosperm order Nymphaeales has been of considerable interest to taxonomists. In general, the view of monocots as a well-defined monophyletic unit derived from within the paraphyletic group of basal dicots [50] is one of the morphology-based theories that are most readily supported by molecular data. Extensive molecular phylogenetic studies have allowed only one refinement to the classical circumscription of monocots, with a total complement of 65000 species and 3000 genera [51]. Specifically, the family Hydatellaceae (twelve species in a single genus) [52] has been transferred from monocots to the early-divergent angiosperms. In the present study our data supported this finding with aid of ITS2 secondary structure alignment, Bayesian network, supermatrix trees from concatenated different loci (ITS2+matk) (Figures 1, 2, 3, 6), analysis of *matK *dataset and molecular clock MP trees (Figure 4). For the first time we have used an extensive molecular morphometrics phylogeny to support Hydatellaceae as a sister group to Nymphaeales.

Besides, the genera *Nuphar *emerges as a monophyletic group with all the *Nuphar *species forming a single cluster with well supported boot strap values. *Nuphar *takes the middle position between these two genera. However, according to the molecular data *Nuphar *(possessing many specialised synapomorphic features) is basal in the clade, thus making *Victoria *and *Nymphaea *closer to each other [53].

However, despite the high amount of characters sampled, the monophyly of the Nymphaeaceae is not convincingly supported. More strikingly, the present data set does not give support for the monophyly of the genus *Nymphaea*. *Nymphaea alba *emerges out group to the order Nymphaeales based on the molecular data as well secondary structure data (Figures 1, 2, 3, 5 and 6) and had a parallel evolution with other representatives from the genus *Nuphar*. In contrast to all previous phylogenetic studies and classifications, it is inferred to be paraphyletic with respect to the *Victoria-Euryale *clade and to *Ondinea*. A reason for the scarcity of informative characters at the base of Nymphaeales could be a rapid, early diversification into the three major lineages. Our results support the opinion that a high rate of evolution within this taxon can be explained by the rapid specialisation of these plants for stepwise adaptation to the aquatic environment.

The other objective in our study was to generate motifs for barcode designing. *matK *genes yielded unique motif regions and thus may provide more variations than other regions in the plant chloroplast genomes. The nr Plant database from European Molecular Biology laboratory (EMBL) was used to test for unique species-specific barcodes that could be used for a species level identification. For this, the sequence belonging to each species was retrieved from the database and used as a query sequence. If the query sequence returned an exact match only to itself, this was scored as a positive identification at the species level. If the query sequence returned an exact match to itself and other members of the same genus, this was scored as a negative identification at the species level, but a positive identification to the genus level. For BLAST, an additional constraint was added to positively score the identification at genus level i.e., the best match as well as the next most similar sequence had to match the genus of the query sequence. If any other genus was included in the top two hits, the result was not considered genus specific. The results are exemplary in the current scenario of plant barcoding. We have reported unique genus specific motif regions in the Order Nymphaeles from *matK *dataset which can be further validated for barcoding and designing of PCR primers.

## Conclusions

The increased application of molecular data in plant systematics has led to an avalanche of sequence profiles flooding the public domain. With a judicious use of these data as phylogenetic signals, the goal of finding universal primer pairs for studying plant genomes won't be troublesome anymore. The unique motifs reported may further be validated for designing barcodes. With Nymphaeales as a case study, it is quite surprising to observe how stepwise adaptation to an aquatic life style has had an impact on water lilies evolution, with the generation of morphological complexity. For the first time we have reported an ITS2 secondary structure alignment and a phylogeny based on the molecular morphometrics that strongly congrued with the current placement of the family Hydatellaceae within the early-divergent angiosperm order Nymphaeales. Though we are far off from completely understanding the selective forces behind these transformations, nevertheless, the phylogenetic signals belied in the comparatively small marker datasets imbibes a source of inspiration to broaden our views on water lily origin and evolution in time.

## Methods

### Taxon sampling and sequence analysis

The dataset used in the present study comprises 64 *matK *and 67 ITS2 sequences from species representing the three families Cabombaceae, Nymphaeaceae and Hydatellaceae of the order Nymphaeales retrieved from GenBank [54] (via Ebot http://www.ncbi.nlm.nih.gov/Class/PowerTools/eutils/ebot/ebot.cgi, an open source interactive tool that generates a Perl script implementing an E-utility pipeline for retrieving large datasets from National Centre for Biotechnology Information (NCBI) with key words and boolean operators. Information on all the species along with GenBank accessions, sequence length and AT, GC content of both the markers are summarized in (Additional Files 5). The sequences were subjected to alignment and manual editing by clustalW [21] and were concatenated for generating a supermatrix using FAsconcat [22]. Subsequently the concatenated files were subjected to mesquite for various file format conversions to be readable by ML and Bayesian methods.

### Phylogenetic reconstruction

The supermatrix dataset (*matK *+ ITS2) covering the Order Nymphaeales were first analysed separately through ML and Bayesian inference [55]. MP analyses were conducted with PhyMl 3.0 [27]. Node support was substantiated through aLRT and bootstrapping.

For Bayesian inference [55] the best models of molecular evolution were determined with aid of MrModeltest version 2.2 [56]. Hence, a Bayesian analysis using MrBayes [23] was carried out for tree construction using a general time reversible substitution model (GTR) with substitution rates estimated by MrBayes. Metropolis-Coupled Markov Chain Monte Carlo (MCMCMC) sampling was performed with two incrementally heated chains that were combinatorially run for 20,000 generations. The convergence of MCMCMC was then monitored by examining the value of the marginal likelihood through generations. Coalescence of substitution rate and rate model parameters were also examined. Average standard deviation of split frequencies was checked and the generations were kept on adding until the standard deviation value was below 0.01. For analysis we ran 10,000,000 generations with a sample frequency of 1000. The values slightly differed because of stochastic effects. The sample of substitution model parameters and samples of trees and branch lengths were summarized by the "sump burnin" and "sumt burnin" commands, respectively. The values in the following commands were adjusted as per the 25% of our samples. A cladogram with the posterior probabilities for each split and a phylogram with mean branch lengths were generated and subsequently read by Mesquite [30]. An alternative method using network analysis was performed using SplitsTree4 [31] with the variable positions in the aligned supermatrix dataset. The alignment file was converted to nexus with READSEQ [57] at Eurpean Bioinformatics Institute (EBI) server readable by SplitsTree4 [31].

### Estimation of molecular clock rates

The molecular clock test was performed by comparing the ML value for the given topology with and without the molecular clock constraints under Jukes-Cantor model [32]. The null hypothesis of equal evolutionary rate throughout the tree was rejected at a 5% significance level (P < 1.20575200719741E-58). The analysis involved 64 *matK *sequences and was computed using MEGA5 [58].

### Analysis of synonymous and non-synonymous substitution rates

Non-synonymous mutations to a DNA sequence cause a change in the translated amino acid sequence, whereas synonymous mutations do not. The comparison between the number of non-synonymous mutations (dn or Ka), and the number of synonymous mutations (ds or Ks), can suggest whether, at the molecular level, natural selection is acting to promote the fixation of advantageous mutations (positive selection) or to remove deleterious mutations (purifying selection). In general, when positive selection dominates, the Ka/Ks ratio is greater than 1; in this case, diversity at the amino acid level is favoured, likely due to the fitness advantage provided by the mutations. Conversely, when negative selection dominates, the Ka/Ks ratio is less than 1; in this case, most amino acid changes are deleterious and, therefore, are selected against. When the positive and negative selection forces balance each other, the Ka/Ks ratio is close to 1. The dS/dN ratio was computed on *matK *sequences only in MEGA5 [58] for testing positive and purifying selection hypothesis.

### Motif identification and testing

The *matK *sequence motifs were identified from aligned sequences using the PRATT software [59]. Besides, the dataset in fasta format were fed to MEME [33] for determining highly significant motifs without any gaps and patterns with variable length gaps if any, were split by MEME into one or more separate motifs. The motif sites were listed in order of increasing statistical significance (p-value) [35]. The p-value of a site is computed from the match score of the site with Position Specific Scoring Matrix (PSSM) for the motif. Further individual datasets for *Nymphaea *and *Nuphur *were subjected to MEME for analyzing the best motifs. The MEME output is subsequently analyzed by MAST [34] for depicting the best scoring matches and similarity to other motifs. The match score are computed if the match completely fits within the sequence and are reported in terms of P-value of the match. MAST takes into account four types of events for calculating the P-value namely the position P-value, sequence P-value, combined P-value and the E-value [35].

### ITS2 secondary structure prediction and analysis

RNA secondary structure prediction for ITS2 sequences were carried out in MATLAB 2012a rnafold [60] and rnaplot [61] functions that uses the nearest-neighbor model and minimizes the total free energy associated with an RNA structure. The minimum free energy was estimated by summing individual energy contributions from base pair stacking, hairpins, bulges, internal loops and multi-branch loops. The energy contributions of these elements are sequence- and length-dependent and have been experimentally determined. The rnafold function uses the nearest-neighbor thermodynamic model to predict the minimum free-energy secondary structure of an RNA sequence. More specifically, the algorithm implemented in rnafold was used for dynamic programming to compute the energy contributions of all possible elementary substructures and then the secondary structures were predicted by considering the combination of elementary substructures whose total free energy were minimum. In this computation, the contribution of coaxially stacked helices is not accounted for, and the formation of pseudoknots (non-nested structural elements) is forbidden. Rnaplot (RNA2ndStruct) was used for drawing RNA secondary structures with specified format values 'Mountain' for ITS2. The secondary structures were computed in form of mountain graphs in MATLAB R2012a environment.

Besides, consensus structures of ITS2 regions were predicted using the RNAz server and LocARNA from Freiburg RNA tools server that outputs a multiple alignment together with a consensus structure. For the folding a very realistic energy model for RNAs was used that features RIBOSUM-like similarity scoring and realistic gap cost. The high performance of LocARNA [38] was mainly achieved by employing base pair probabilities during the alignment procedure. Results of the various species were compared to unravel the folding pattern common to them all for establishing the conserved structural models across several genera of Nymphaeales using 4SALE [39] and subsequently incorporated in ProfDistS [40] for generating molecular morphometrics phylogeny. The ProfDistS [40] output was read by NjPlot [41].

## Competing interests

The authors declare that they have no competing interests.

## Authors' contributions

PT and DKB conceived of the study and participated in its design, coordination and manuscript writing. DKB performed the computational analysis, participated in the design of the study and manuscript preparation. MD participated in the computational analysis and literature screening. SK carried out the secondary structure analysis and developed perl scripts for the present study. All authors have read and approved the final manuscript.

## Supplementary Material

Additional file 1 **Concatenated aligned Supermatrix dataset of ITS2 and matK generated using FASconCAT version 1.0**. The concatenated aligned supermatrix file is in nexus format and can be viewed in Mesquite. Click here for file

Additional file 2 **Codon-based Test of Positive Selection (dS/dN) analysis for matK sequences**. Click here for file

Additional file 3 **Codon-based Test of Purifying Selection (dS/dN) analysis for matK sequences**. Click here for file

Additional file 4 **Top scoring unique motif sequence matches shown for each of the matK sequences in the order Nymphaeales**. Click here for file

Additional file 5 **Nucleotide composition and GC content of ITS2 sequences of Nymphaeales**. Click here for file

Additional file 6 **Consensus alignment of ITS2 sequences showing conserved regions for secondary structure prediction across Nymphaeales**. The three families are represented by the genera *Brasenia*, *Cabomba*, *Euryale*, *Nuphur*, *Nymphaea*, *Victoria *and *Trithuria*. Standard nucleotide ambiguity codes are used. Click here for file

Additional file 7 **Overall summary of secondary structures for ITS2 multiple alignment of Nymphaeales (*Brasenia, Cabomba*, *Euryale*, *Nuphur*, *Nymphaea *and *Victoria*) showing detailed information (z-score, structure conservation index, RNAz P-value, etc.) along with a Dot Plot graph**. Click here for file

Additional file 8 **Matlab generated mountain graph plots of Nymphaeales (ITS2 sequences)**. Click here for file

Additional file 9 Nucleotide composition and GC content of matK sequences of Nymphaeales Click here for file
