# Supplementary figures and images for: Phylogenetic reconstruction in the Order Nymphaeales: ITS2 secondary structure analysis and in silico testing of maturase k (matK) as a potential marker for DNA bar coding
Source: BMC Bioinformatics. 2012 Dec 7;13(Suppl 17):S26. doi: 10.1186/1471-2105-13-S17-S26 (PMC3521246; doi:10.1186/1471-2105-13-S17-S26)

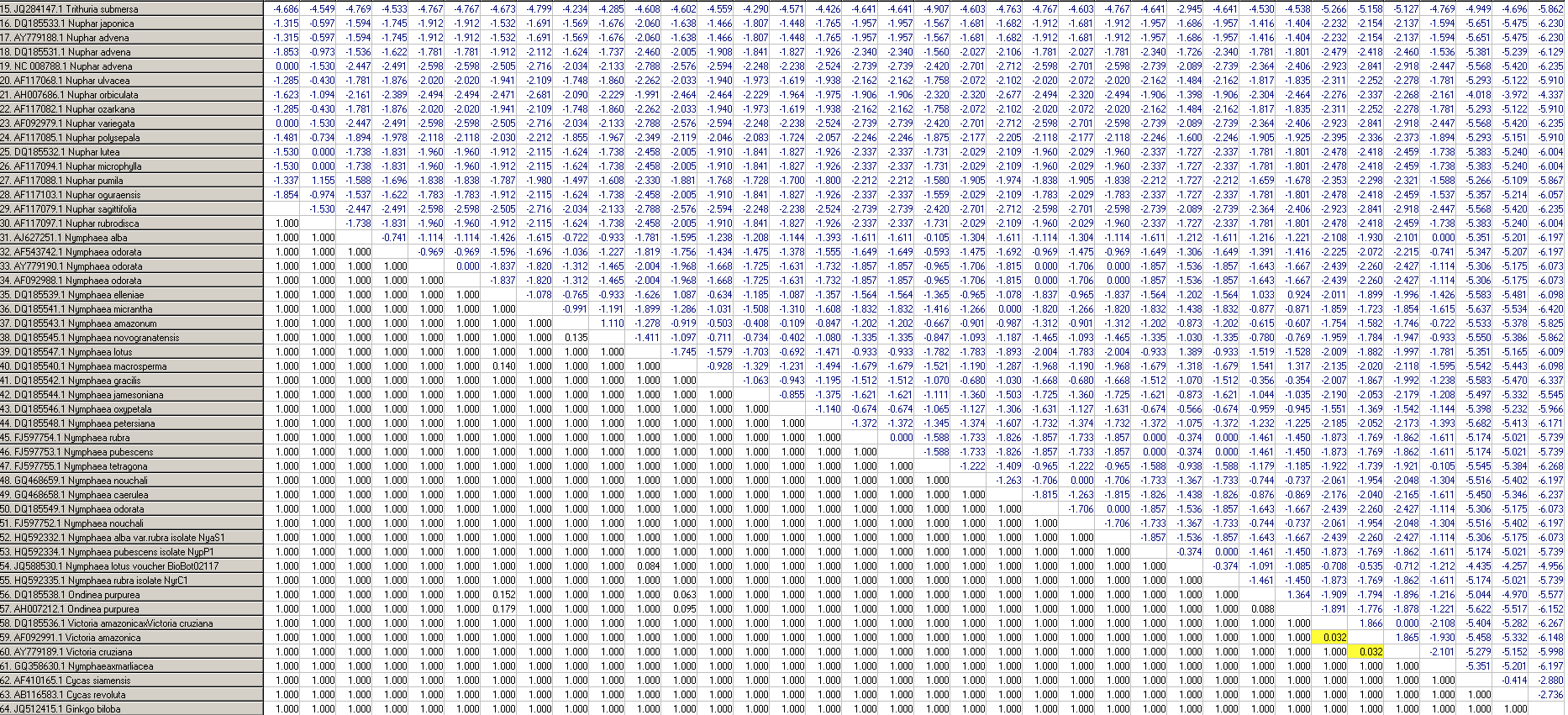

Supplement: Additional file 2 — Codon-based Test of Positive Selection (dS/dN) analysis for matK sequences. [file 1471-2105-13-S17-S26-S2.PNG]

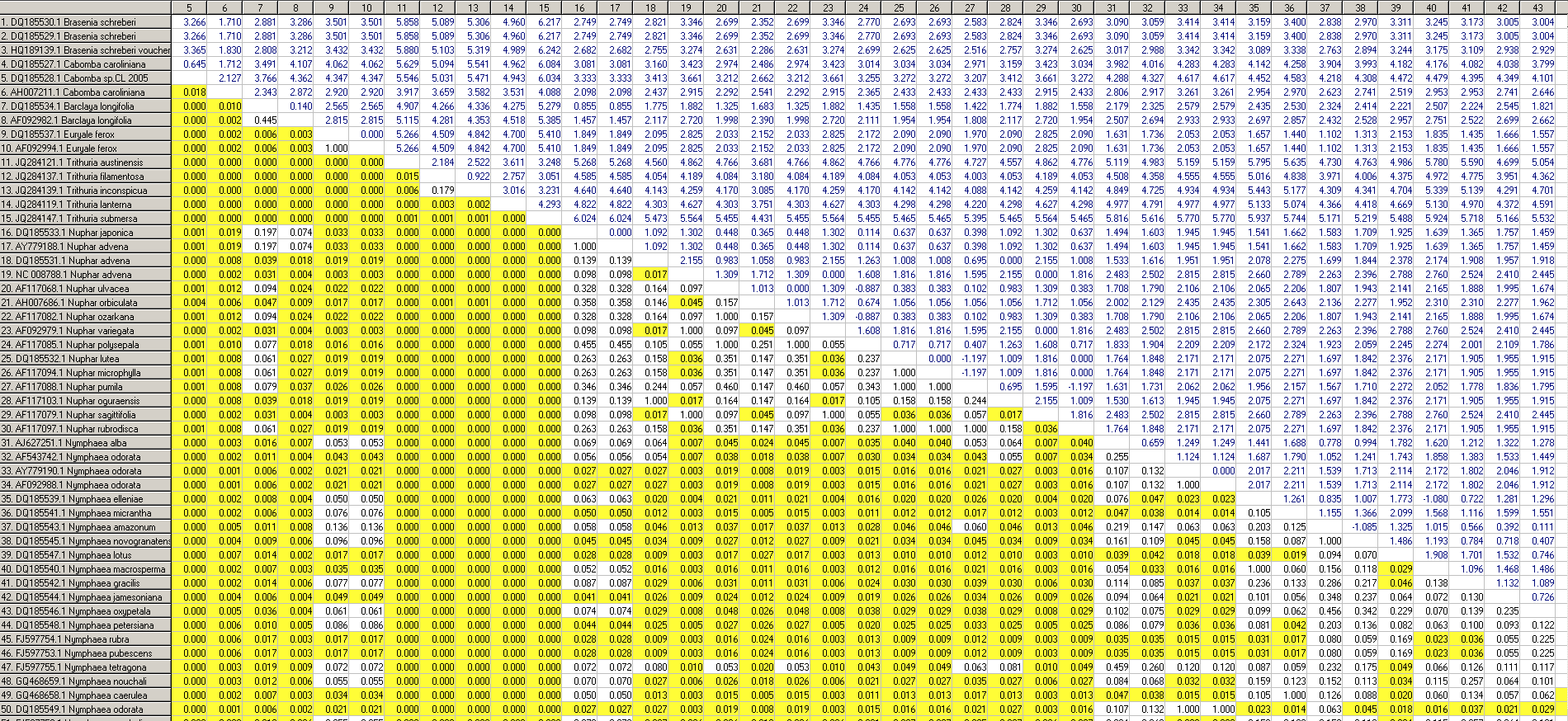

Supplement: Additional file 3 — Codon-based Test of Purifying Selection (dS/dN) analysis for matK sequences. [file 1471-2105-13-S17-S26-S3.PNG]
