# Supplementary material for: Phylogenetic reconstruction in the Order Nymphaeales: ITS2 secondary structure analysis and in silico testing of maturase k (matK) as a potential marker for DNA bar coding
Source: BMC Bioinformatics. 2012 Dec 7;13(Suppl 17):S26. doi: 10.1186/1471-2105-13-S17-S26 (PMC3521246; doi:10.1186/1471-2105-13-S17-S26)
Supplement: Additional file 4 — Top scoring unique motif sequence matches shown for each of the matK sequences in the order Nymphaeales. [file 1471-2105-13-S17-S26-S4.DOCX]

**Table S1: Top scoring unique motif sequence matches shown for each of the matK sequences.**

| Genus | **Motif** | **Width** | **Best possible match** | | **Similarity** | | | ***E*-value** | ***p*-value** |
| --- | --- | --- | --- | --- | --- | --- | --- | --- | --- |
|  |  |  | **(+)** | **(-)** | **Motif 1** | **Motif 2** | **Motif 3** |  |  |
| Brasenia | Motif 1 | 60 | GTAGTGGCGCCTCGTAATGATTCTCAAAGGACCCTGCCCCTCTGGTTCTTCAAGGAACCT | AGGTTCCTTGAAGAACCAGAGGGGCAGGGTCCTTTGAGAATCATTACGAGGCGCCACTAC | - | 0.17 | 0.17 | 2.8e-93 | 4.42e-95 |
|  | Motif 2 | 60 | ACCCTACCCAATACATCTGGAAATCTTGCTTCAAACTCTTCGTACCCGGATACGAGATGC | GCATCTCGTATCCGGGTACGAAGAGTTTGAAGCAAGATTTCCAGATGTATTGGGTAGGGT | 0.17 | - | 0.23 | 8.7e-93 | 1.36e-94 |
|  | Motif 3 | 60 | CGAAATTCTGTAACGTATCAGGGCATCCTACTAGTAAGTCAGTCTGGGCTGATTTGTCGG | CCGACAAATCAGCCCAGACTGACTTACTAGTAGGATGCCCTGATACGTTACAGAATTTCG | 0.17 | 0.23 | - | 8.7e-93 | 1.36e-94 |
| Cabomba | Motif 1 | 60 | AAGCGTCTTGTAGTGGCACCTCGTAATGATTCTCAAAGGACCCTGCCCCTCTGGTTCTTC | GAAGAACCAGAGGGGCAGGGTCCTTTGAGAATCATTACGAGGTGCCACTACAAGACGCTT | - | 0.12 | 0.17 | 2.5e-93 | 3.85e-95 |
|  | Motif 2 | 60 | ACCCTACCCCATACATCTGGAAATCTTGCTTCAAACTCTTCGTACCCGGGTACGAGATGC | GCATCTCGTACCCGGGTACGAAGAGTTTGAAGCAAGATTTCCAGATGTATGGGGTAGGGT | 0.12 | - | 0.13 | 1.6e-92 | 2.49e-94 |
|  | Motif 3 | 60 | ACTAGAGGGTTTTGCAGGAATCATGGAAATTGCATTCTCGACGCGATTAGTATCTTCTCT | AGAGAAGATACTAATCGCGTCGAGAATGCAATTTCCATGATTCCTGCAAAACCCTCTAGT | 0.17 | 0.13 | - | 1.5e-91 | 2.40e-93 |
| Barclaya | Motif 1 | 59 | CCCCACCCAATCCATTTGGAAATCTTGCTTCAAACTCTTCGCACCCGGATACGAGATGC | GCATCTCGTATCCGGGTGCGAAGAGTTTGAAGCAAGATTTCCAAATGGATTGGGTGGGG | - | 0.20 | 014 | 3.2e-91 | 5.04e-93 |
|  | Motif 2 | 60 | CGCTTCGTAATGATTCTCAAAGGACCCTGCCCCTCTGGTTCCTCAAGGAACCTTTGATGC | GCATCAAAGGTTCCTTGAGGAACCAGAGGGGCAGGGTCCTTTGAGAATCATTACGAAGCG | 0.20 | - | 0.14 | 6.3e-91 | 9.79e-93 |
|  | Motif 3 | 58 | GTCAGTCTGGGCCGATTCGTCGGATTCTGATATTATTGCTCGATTCGGGTGGATATGC | GCATATCCACCCGAATCGAGCAATAATATCAGAATCCGACGAATCGGCCCAGACTGAC | 0.14 | 0.14 | - | 5.1e-83 | 8.03e-85 |
| Euryale | Motif 1 | 60 | CGCCTCGTAATGATTCTCAAAGGACCCTGCCCCTCTGGTTCTTCAAGGAACCTTTGATGC | GCATCAAAGGTTCCTTGAAGAACCAGAGGGGCAGGGTCCTTTGAGAATCATTACGAGGCG | - | 0.14 | 0.16 | 2.2e-92 | 3.46e-94 |
|  | Motif 2 | 60 | GTCAGTCTGGGCCGATTCGTCGGATTCTGATATTATTGGTCGATTCGGGTGGATATGCAG | CTGCATATCCACCCGAATCGACCAATAATATCAGAATCCGACGAATCGGCCCAGACTGAC | 0.14 | - | 0.16 | 2.2e-92 | 3.46e-94 |
|  | Motif 3 | 59 | CCCTACCCAATCCATCTGGAAATCTTGCTTCAAACTCTTCGCACTCGGATACGAGATGC | GCATCTCGTATCCGAGTGCGAAGAGTTTGAAGCAAGATTTCCAGATGGATTGGGTAGGG | 0.16 | 0.16 | - | 3.9e-90 | 6.06e-92 |
| Nuphar | Motif 1 | 60 | GTAGTGGCACCTCGTAATGATTCTCAAAGGACCCTGCCCCTCTGGTTCTTCAAGGAACCC | GGGTTCCTTGAAGAACCAGAGGGGCAGGGTCCTTTGAGAATCATTACGAGGTGCCACTAC | - | 0.17 | 0.17 | 1.4e-92 | 2.19e-94 |
|  | Motif 2 | 60 | AGGTTCGTTGGGCCCAACAGGAGTTTTTATTCTCAAACGATATCAGAGGGTTTTGCAGGA | TCCTGCAAAACCCTCTGATATCGTTTGAGAATAAAAACTCCTGTTGGGCCCAACGAACCT | 0.17 | - | 0.17 | 1.6e-92 | 2.52e-94 |
|  | Motif 3 | 60 | CCCGGATACGAGATGCTCCTTCTTTGCATTTATTGAGATGCTTTCTACACGAGCATCATA | TATGATGCTCGTGTAGAAAGCATCTCAATAAATGCAAAGAAGGAGCATCTCGTATCCGGG | 0.17 | 0.17 | - | 1.6e-92 | 2.52e-94 |
| Nymphaea | Motif 1 | 60 | CGCCTCGTAATGATTCTCAAAGGACCCTGCCCCTCTGGTTCTTCAAGGAACCTTTGATGC | GCATCAAAGGTTCCTTGAAGAACCAGAGGGGCAGGGTCCTTTGAGAATCATTACGAGGCG | - | 0.17 | 0.16 | 6.1e-93 | 9.59e-95 |
|  | Motif 2 | 60 | CTGGGCTATCTTTCAGGTGTACGACTAACGCCTTGGGTGATAAGGAGTCAAATGCTAGAG | CTCTAGCATTTGACTCCTTATCACCCAAGGCGTTAGTCGTACACCTGAAAGATAGCCCAG | 0.17 | - | 0.15 | 6.2e-93 | 9.71e-95 |
|  | Motif 3 | 60 | GTCAGTCTGGGCCGATTCGTCGGATTCTGATATTATTGCTCGATTCGGGTGGATATGCAG | CTGCATATCCACCCGAATCGAGCAATAATATCAGAATCCGACGAATCGGCCCAGACTGAC | 0.16 | 0.15 | - | 6.2e-93 | 9.71e-95 |
| Ondinea | Motif 1 | 60 | CGCCTCGTAATGATTCTCAAAGGACCCTGCCCCTCTGGTTCTTCAAGGAACCTTTGATGC | GCATCAAAGGTTCCTTGAAGAACCAGAGGGGCAGGGTCCTTTGAGAATCATTACGAGGCG | - | 0.16 | 0.14 | 1e-92 | 1.60e-94 |
|  | Motif 2 | 59 | CCCTACCCAATCCATCTGGAAATCTTGCTTCAAACTCTCCGCACTCGGATACGAGATGC | GCATCTCGTATCCGAGTGCGGAGAGTTTGAAGCAAGATTTCCAGATGGATTGGGTAGGG | 0.16 | - | 0.14 | 1.8e-92 | 2.88e-94 |
|  | Motif 3 | 60 | GTCAGTCTGGGCCGATTCGTCGGATTCTGATATTATTGCTCGATTCGGGTGGATATGCAG | CTGCATATCCACCCGAATCGAGCAATAATATCAGAATCCGACGAATCGGCCCAGACTGAC | 0.14 | 0.14 | - | 1.8e-92 | 2.88e-94 |
| Victoria | Motif 1 | 60 | GTCAGTCTGGGCCGATTCGTCGGATTCTGATATTATTGCTCGATTCGGGTGGATATGCAG | CTGCATATCCACCCGAATCGAGCAATAATATCAGAATCCGACGAATCGGCCCAGACTGAC | - | 0.14 | 0.23 | 1.1e-92 | 1.67e-94 |
|  | Motif 2 | 60 | ACCCTACCCAATCCATCTGGAAATCTTGCTTCAAACTCTTCGCACTCGGATACGAGATGC | GCATCTCGTATCCGAGTGCGAAGAGTTTGAAGCAAGATTTCCAGATGGATTGGGTAGGGT | 0.14 | - | 0.21 | 1.1e-92 | 1.69e-94 |
|  | Motif 3 | 60 | AAGGACCCTGCCCCTCTGGTTCTTCAAGGAACCTTTGATGCATTATGTTAGGTATCAAGG | CCTTGATACCTAACATAATGCATCAAAGGTTCCTTGAAGAACCAGAGGGGCAGGGTCCTT | 0.23 | 0.21 | - | 1.1e-92 | 1.69e-94 |
| Trithuria | Motif 1 | 60 | CCCCATTAATCCTGAAATCTTCGTTCAGACTCTTCGAGGCTGCATACGGGATGCTCCTTC | GAAGGAGCATCCCGTATGCAGCCTCGAAGAGTCTGAACGAAGATTTCAGGATTAATGGGG | - | 0.20 | 0.13 | 1.7e-92 | 2.67e-94 |
|  | Motif 2 | 60 | TGTAGGAGTTCTTCCTAATGACTTTCAAAGGTACCTTTACCTATGGGTTAGCAAAGAACC | GGTTCTTTGCTAACCCATAGGTAAAGGTACCTTTGAAAGTCATTAGGAAGAACTCCTACA | 0.20 | - | 0.21 | 2.4e-92 | 3.77e-94 |
|  | Motif 3 | 60 | GGGCTATCTTTCAGGTGTACAATTTACTCCTTGGGTGGTAAGAAGTCAAATGCTAGAGAG | CTCTCTAGCATTTGACTTCTTACCACCCAAGGAGTAAATTGTACACCTGAAAGATAGCCC | 0.13 | 0.21 | - | 2.4e-92 | 3.77e-94 |
