# Supplementary material for: Phylogenetic reconstruction in the Order Nymphaeales: ITS2 secondary structure analysis and in silico testing of maturase k (matK) as a potential marker for DNA bar coding
Source: BMC Bioinformatics. 2012 Dec 7;13(Suppl 17):S26. doi: 10.1186/1471-2105-13-S17-S26 (PMC3521246; doi:10.1186/1471-2105-13-S17-S26)
Supplement: Additional file 5 — Nucleotide composition and GC content of ITS2 sequences of Nymphaeales. [file 1471-2105-13-S17-S26-S5.DOCX]

**Table S2: Nucleotide composition and GC content of ITS2 sequences of Nymphaeales.**

| **Sl. No.** | **Species** | **Accession number** | **Length** | **%GC** | **A** | **T** | **G** | **C** |
| --- | --- | --- | --- | --- | --- | --- | --- | --- |
| 1 | *Brasenia schreberi* | HQ189142.1 | 217 | 64.5 | 37 | 40 | 72 | 68 |
| 2 | *Brasenia schreberi* | HQ189140.1 | 217 | 65 | 37 | 39 | 72 | 69 |
| 3 | *Brasenia schreberi* | AB022738.1 | 213 | 64.3 | 36 | 40 | 70 | 67 |
| 4 | *Brasenia schreberi* | FM242141.1 | 113 | 69 | 14 | 21 | 40 | 38 |
| 5 | *Cabomba furcata* | JF805749.1 | 245 | 62.4 | 39 | 53 | 84 | 69 |
| 6 | *Cabomba caroliniana* | JF805747.1 | 246 | 58.1 | 47 | 56 | 82 | 61 |
| 7 | *Cabomba sp NY401* | FM242143.1 | 190 | 53.2 | 39 | 50 | 65 | 36 |
| 8 | *Barclaya longifolia* | FM242140.1 | 245 | 66.1 | 28 | 55 | 89 | 73 |
| 9 | *Euryale ferox* | AF136287.1 | 202 | 44.1 | 54 | 59 | 58 | 31 |
| 10 | *Euryale ferox* | FM242144.1 | 211 | 43.1 | 52 | 68 | 55 | 36 |
| 11 | *Trithuria austinensis* | JQ284161.1 | 316 | 63.9 | 48 | 66 | 104 | 98 |
| 12 | *Trithuria filamentosa* | JQ284173.1 | 318 | 63.2 | 52 | 65 | 102 | 99 |
| 13 | *Trithuria inconspicua* | JQ284177.1 | 318 | 63.2 | 52 | 65 | 102 | 99 |
| 14 | *Trithuria lanterna* | JQ284159.1 | 311 | 64.3 | 43 | 68 | 108 | 92 |
| 15 | *Trithuria submersa* | JQ284185.1 | 318 | 64.5 | 44 | 69 | 107 | 98 |
| 16 | *Nuphar variegata* | EF526384.1 | 254 | 58.7 | 43 | 62 | 84 | 65 |
| 17 | *Nuphar sinensis* | AF136288.1 | 232 | 51.7 | 47 | 65 | 58 | 62 |
| 18 | *Nuphar microphylla* | AF067598.1 | 239 | 57.7 | 41 | 60 | 80 | 58 |
| 19 | *Nuphar japonica* | AF067596.1 | 239 | 58.2 | 39 | 61 | 82 | 57 |
| 20 | *Nuphar lutea* | AF067594.1 | 240 | 57.9 | 40 | 61 | 81 | 58 |
| 21 | *Nuphar sagittifolia* | AF067591.1 | 239 | 58.6 | 40 | 59 | 81 | 59 |
| 22 | *Nuphar orbiculata* | AF067589.1 | 240 | 58.8 | 40 | 59 | 81 | 60 |
| 23 | *Nuphar advena* | AF067583.1 | 238 | 58.8 | 40 | 58 | 81 | 59 |
| 24 | *Nuphar variegata* | AF067579.1 | 113 | 58.4 | 17 | 30 | 38 | 28 |
| 25 | *Nuphar lutea* | FM242147.1 | 245 | 58.4 | 41 | 61 | 82 | 61 |
| 26 | *Nuphar advena* | FM242145.1 | 251 | 57.8 | 41 | 65 | 82 | 63 |
| 27 | *Nuphar japonica* | FM242146.1 | 245 | 57.6 | 40 | 64 | 83 | 58 |
| 28 | *Nuphar shimadae* | AB022737.1 | 232 | 57.3 | 38 | 61 | 81 | 52 |
| 29 | *Nuphar subintegerrima* | AB022735.1 | 244 | 58.2 | 40 | 62 | 83 | 59 |
| 30 | *Nuphar pumila* | AB022736.1 | 248 | 58.5 | 41 | 62 | 83 | 62 |
| 31 | *Nuphar ulvacea* | AF067587.1 | 239 | 58.6 | 40 | 59 | 81 | 59 |
| 32 | *Nuphar ozarkana* | AF067585.1 | 239 | 58.6 | 40 | 59 | 81 | 59 |
| 33 | *Nuphar polysepala* | AF067577.1 | 239 | 58.6 | 40 | 59 | 81 | 59 |
| 34 | *Nuphar oguraensis* | AF067592.1 | 192 | 56.3 | 33 | 51 | 61 | 47 |
| 35 | *Nuphar rubrodisca* | AF067581.1 | 239 | 58.6 | 40 | 59 | 81 | 59 |
| 36 | *Nymphaea x marliacea* | FJ597739.1 | 227 | 56.8 | 40 | 58 | 70 | 59 |
| **Sl. No.** | **Species** | **Accession number** | **Length** | **%GC** | **A** | **T** | **G** | **C** |
| 37 | *Nymphaea nouchali* | FJ597741.1 | 257 | 50.2 | 51 | 77 | 68 | 61 |
| 38 | *Nymphaea alba var rubra* | GU222363.1 | 233 | 57.1 | 39 | 61 | 74 | 59 |
| 39 | *Nymphaea alba var rubra* | GU222359.1 | 232 | 56.5 | 41 | 60 | 71 | 60 |
| 40 | *Nymphaea rubra* | GQ468655.1 | 236 | 55.9 | 44 | 60 | 70 | 62 |
| 41 | *Nymphaea pubescens* | GQ468653.1 | 244 | 55.7 | 46 | 62 | 72 | 64 |
| 42 | *Nymphaea pubescens* | GQ468652.1 | 243 | 55.6 | 46 | 62 | 72 | 63 |
| 43 | *Nymphaea rubra* | GU199472.1 | 245 | 55.1 | 46 | 64 | 71 | 64 |
| 44 | *Nymphaea rubra* | GU199466.1 | 245 | 56.3 | 46 | 61 | 72 | 66 |
| 45 | *Nymphaea rubra* | GU199462.1 | 245 | 55.5 | 46 | 63 | 71 | 65 |
| 46 | *Nymphaea rubra* | GU199458.1 | 245 | 55.9 | 45 | 63 | 71 | 66 |
| 47 | *Nymphaea tetragona* | GU199475.1 | 231 | 51.5 | 42 | 70 | 67 | 52 |
| 48 | *Nymphaea odorata* | EF526395.1 | 228 | 55.7 | 40 | 61 | 70 | 57 |
| 49 | *Nymphaea odorata subsp tuberosa* | EF526404.1 | 228 | 57.9 | 36 | 60 | 73 | 59 |
| 50 | *Nymphaea nouchali* | FJ597742.1 | 258 | 50.4 | 51 | 77 | 70 | 60 |
| 51 | *Nymphaea alba* | AF136285.1 | 224 | 48.7 | 43 | 72 | 40 | 69 |
| 52 | *Nymphaea x marliacea* | FJ198403.1 | 232 | 56 | 41 | 61 | 71 | 59 |
| 53 | *Nymphaea pubescens* | EU191039.1 | 245 | 55.9 | 46 | 62 | 73 | 64 |
| 54 | *Nymphaea pubescens* | FJ198406.1 | 245 | 55.5 | 46 | 63 | 72 | 64 |
| 55 | *Nymphaea alba var rubra* | EU191036.2 | 234 | 56 | 40 | 63 | 69 | 62 |
| 56 | *Nymphaea lotus* | EU428063.1 | 236 | 55.9 | 42 | 62 | 71 | 61 |
| 57 | *Nymphaea jamesoniana* | FM242152.1 | 234 | 53.4 | 42 | 67 | 71 | 54 |
| 58 | *Nymphaea caerulea* | FJ597738.1 | 257 | 50.2 | 50 | 78 | 68 | 61 |
| 59 | *Nymphaea gracilis* | FM242151.1 | 248 | 52.4 | 44 | 74 | 70 | 60 |
| 60 | *Nymphaea amazonum* | FM242149.1 | 236 | 59.3 | 38 | 58 | 76 | 64 |
| 61 | *Nymphaea nouchali* | GU199474.1 | 257 | 50.2 | 51 | 77 | 68 | 61 |
| 62 | *Ondinea purpurea* | FJ026600.1 | 260 | 46.9 | 55 | 83 | 66 | 56 |
| 63 | *Victoria cruziana* | FM242157 | 194 | 48.5 | 42 | 58 | 56 | 38 |
| 64 | *Victoria amazonica x Victoria cruziana* | FM242158.1 | 196 | 49.0 | 42 | 58 | 56 | 40 |
| 65 | *Cycas siamensis* | AF394442.1 | 251 | 66.9 | 36 | 47 | 88 | 80 |
| 66 | *Cycas revoluta* | AF394434.1 | 250 | 65.6 | 35 | 51 | 85 | 79 |
| 67 | *Ginkgo biloba* | GQ463503.1 | 239 | 56.9 | 50 | 53 | 66 | 70 |
