# Supplementary material for: Phylogenetic reconstruction in the Order Nymphaeales: ITS2 secondary structure analysis and in silico testing of maturase k (matK) as a potential marker for DNA bar coding
Source: BMC Bioinformatics. 2012 Dec 7;13(Suppl 17):S26. doi: 10.1186/1471-2105-13-S17-S26 (PMC3521246; doi:10.1186/1471-2105-13-S17-S26)
Supplement: Additional file 6 — Consensus alignment of ITS2 sequences showing conserved regions for secondary structure prediction across Nymphaeales. The three families are represented by the genera Brasenia, Cabomba, Euryale, Nuphur, Nymphaea, Victoria and Trithuria. Standard nucleotide ambiguity codes are used. [file 1471-2105-13-S17-S26-S6.DOCX]

A


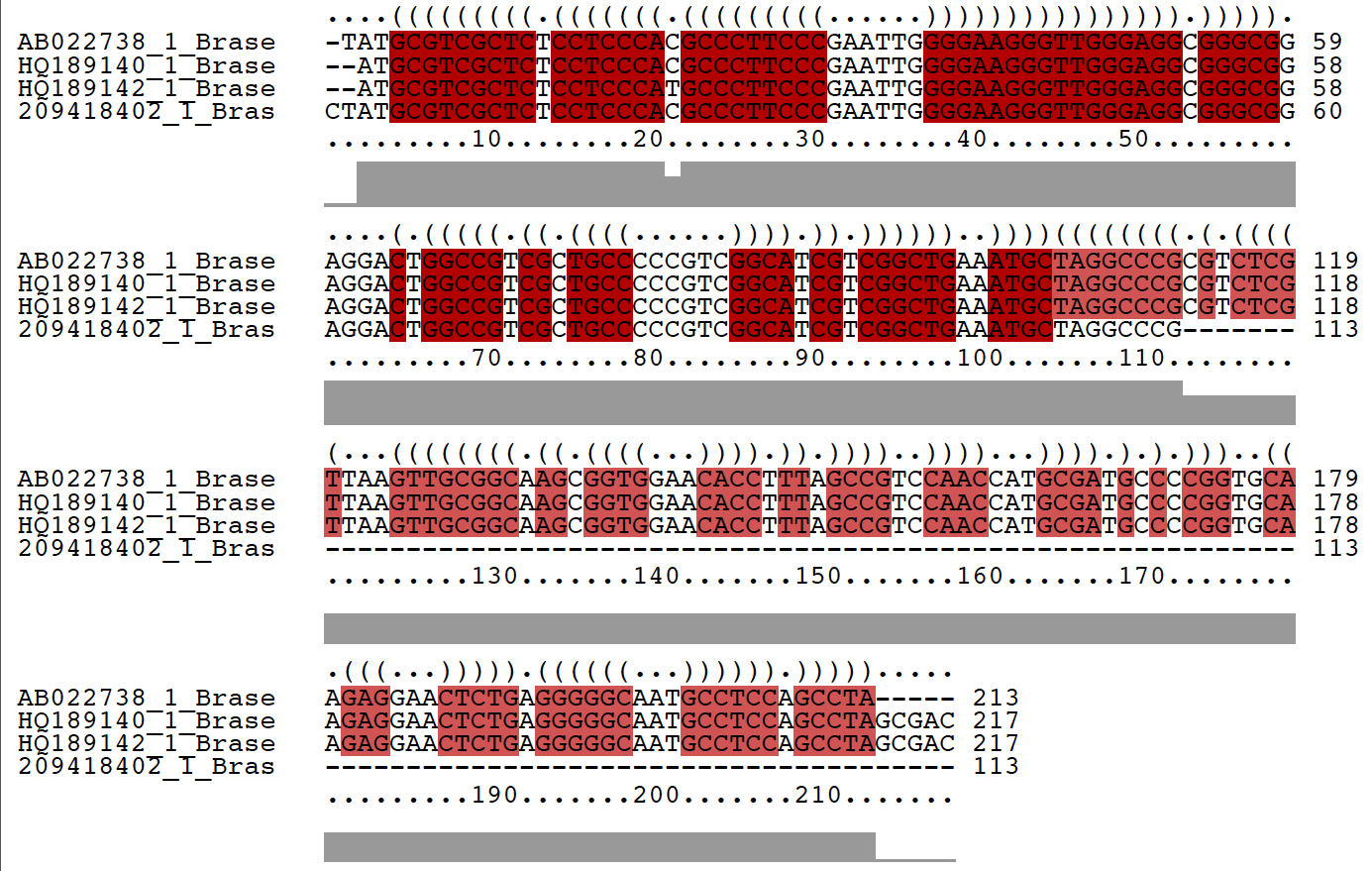


B


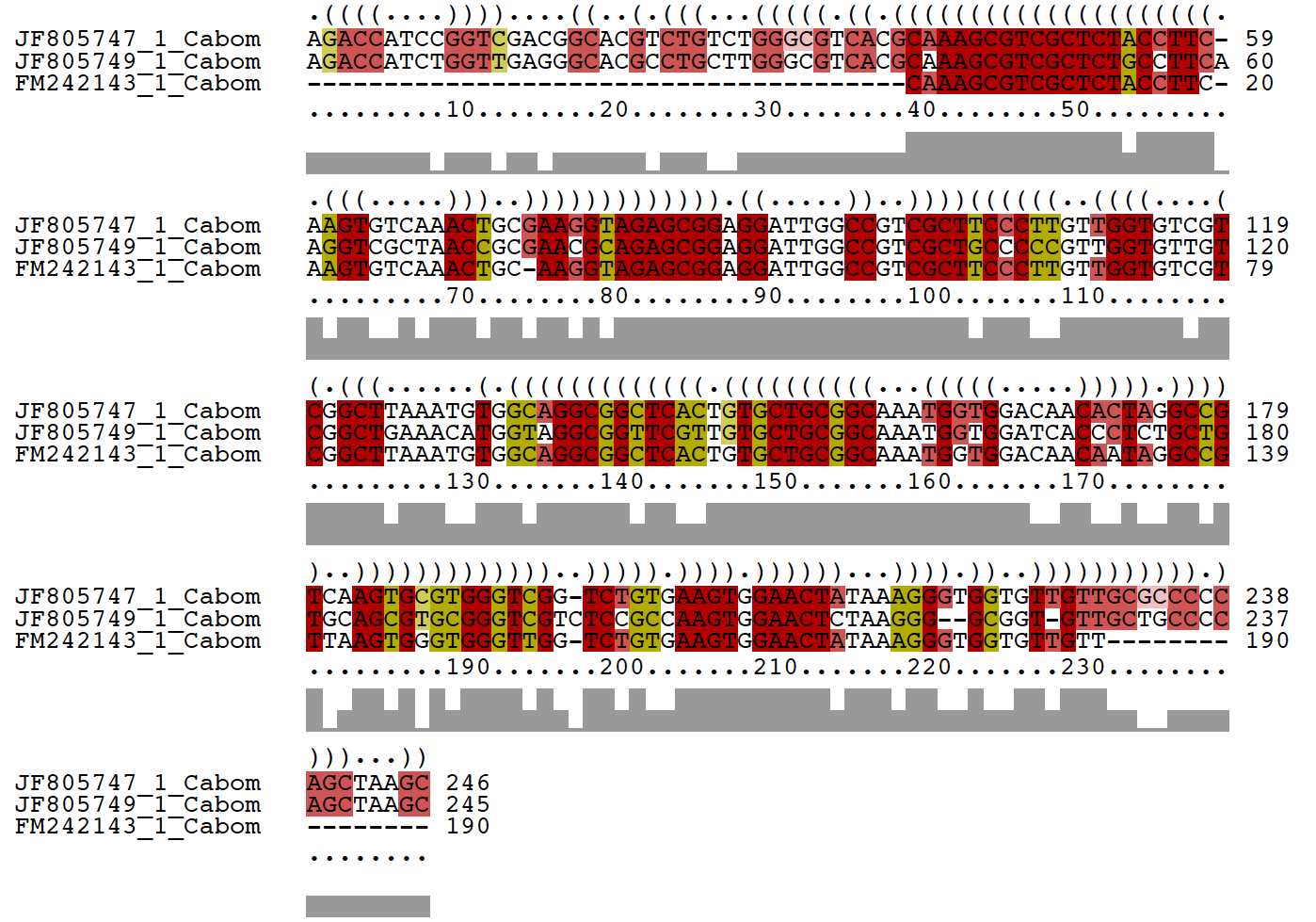


### Figure S1: Consensus alignment of ITS2 sequences showing conserved regions for secondary structure prediction across genus *Brasenia* (A) and *Cabomba* (B). Standard nucleotide ambiguity codes are used.

A


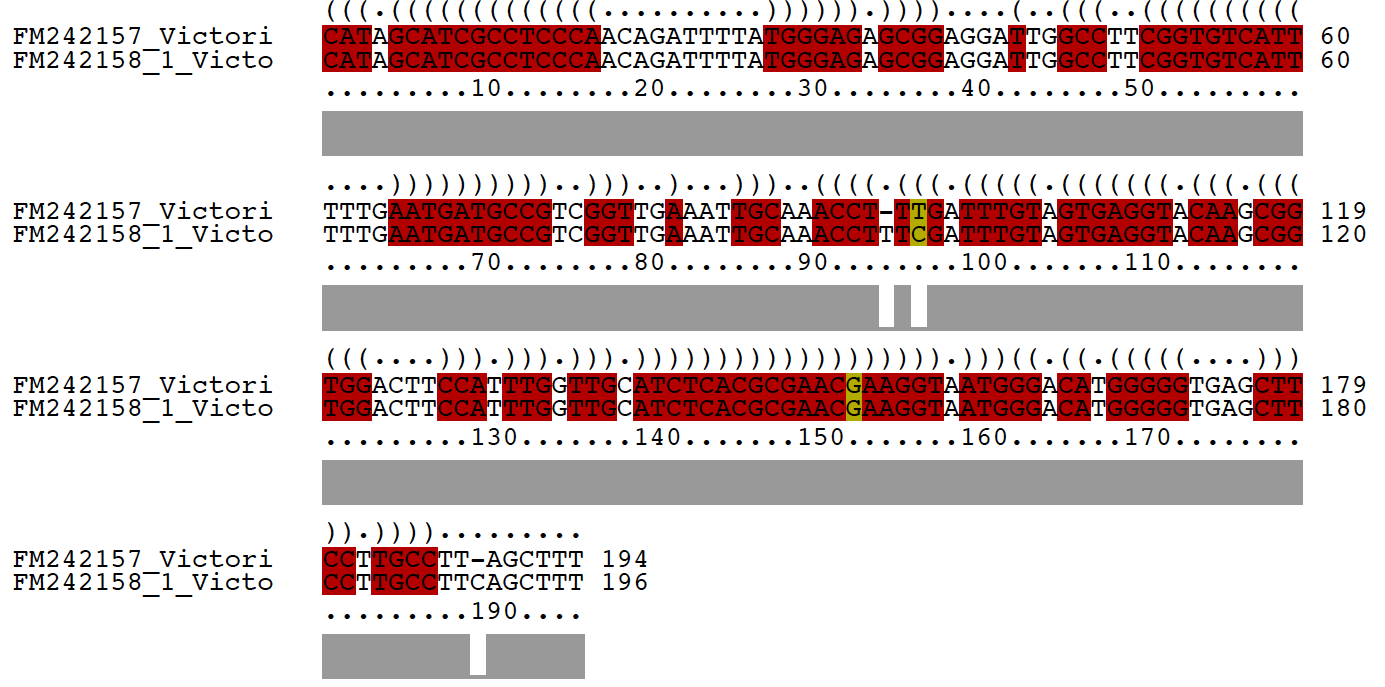

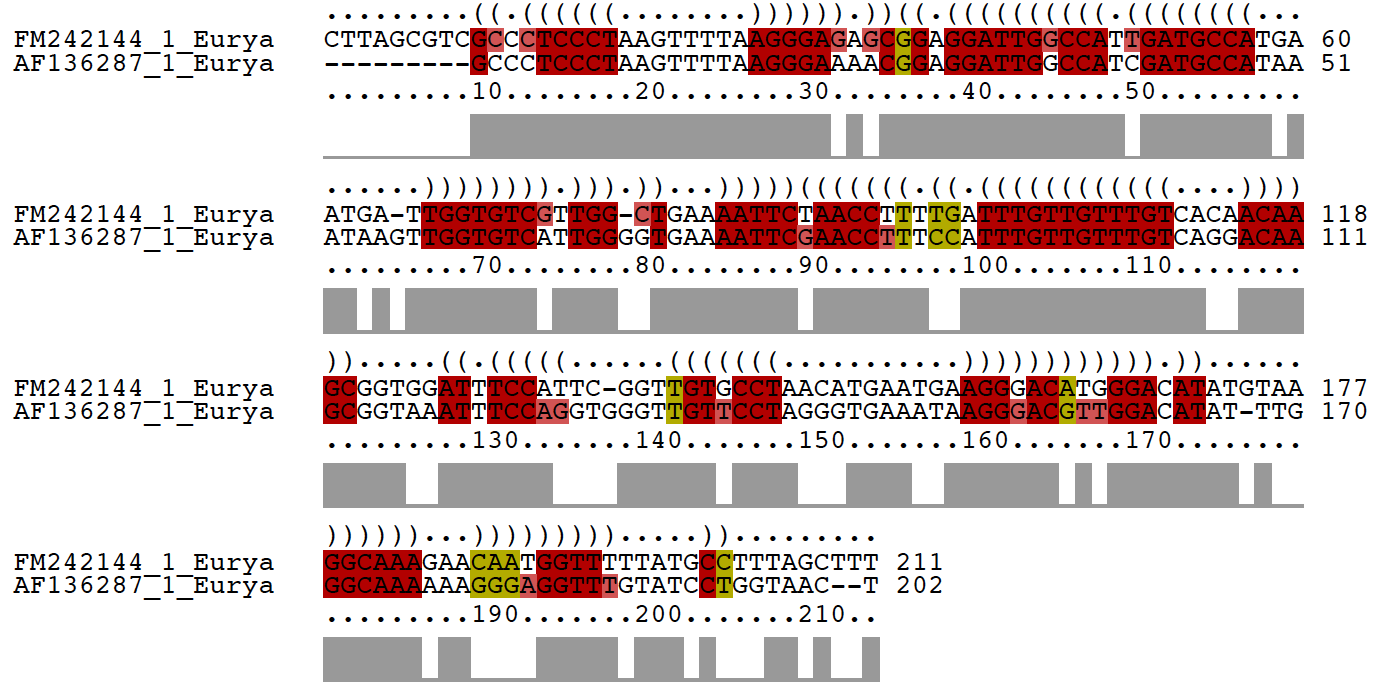


B

### Figure S2: Consensus alignment of ITS2 sequences showing conserved regions for secondary structure prediction across genus *Euryale* (A) and *Nuphur* (B). Standard nucleotide ambiguity codes are used.


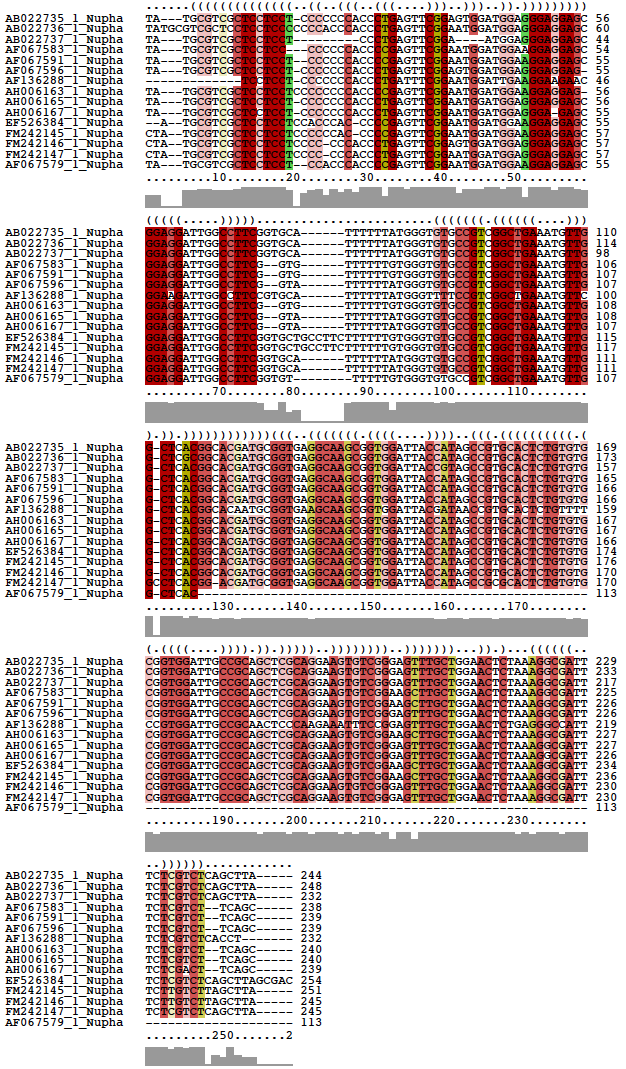


### Figure S3: Consensus alignment of ITS2 sequences showing conserved regions for secondary structure prediction across genus *Nymphaea*. Standard nucleotide ambiguity codes are used.


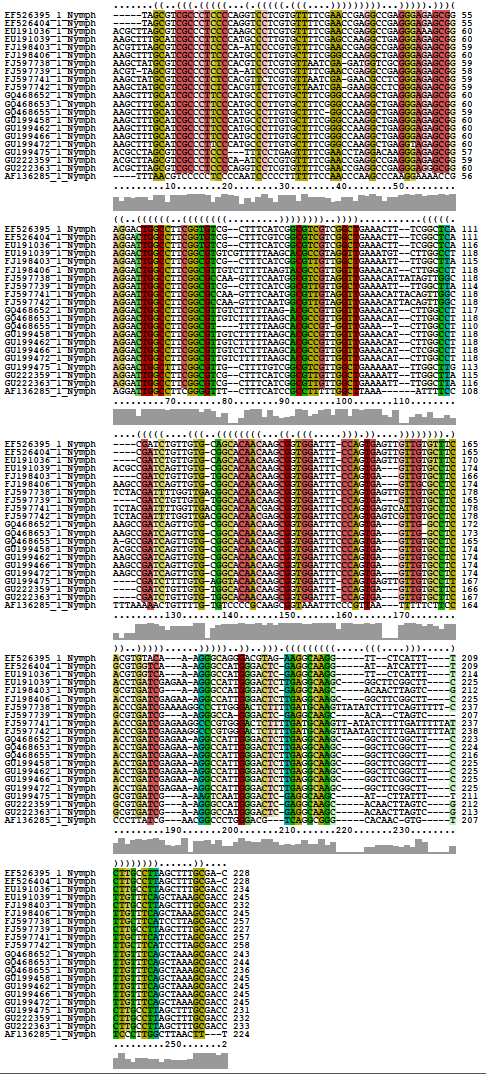


### Figure S4: Consensus alignment of ITS2 sequences showing conserved regions for secondary structure prediction across genus *Victoria*. Standard nucleotide ambiguity codes are used.


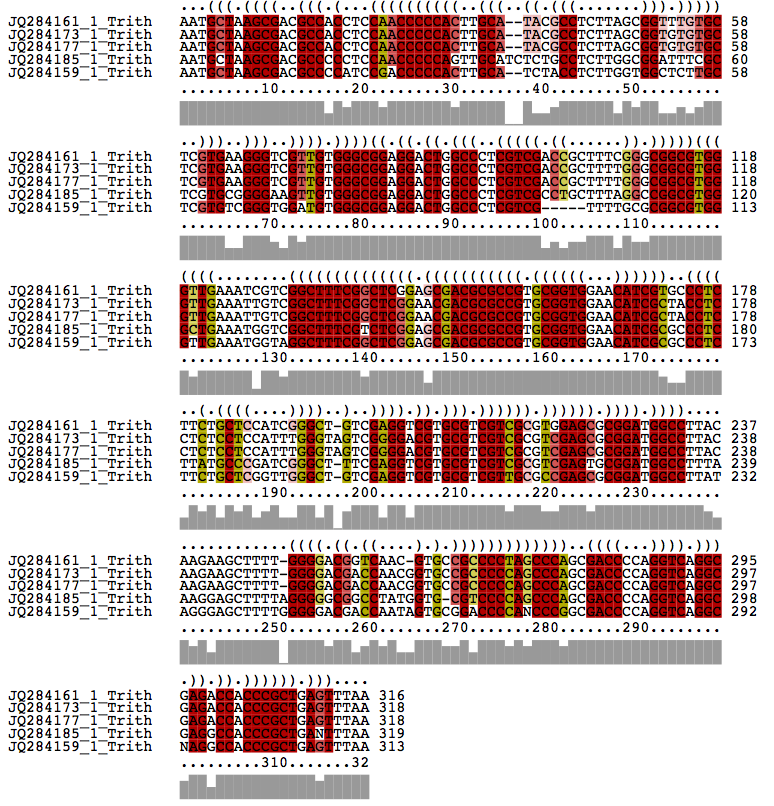


### Figure S5: Consensus alignment of ITS2 sequences showing conserved regions for secondary structure prediction across genus *Trithuria*. Standard nucleotide ambiguity codes are used.
