# Supplementary material for: Phylogenetic reconstruction in the Order Nymphaeales: ITS2 secondary structure analysis and in silico testing of maturase k (matK) as a potential marker for DNA bar coding
Source: BMC Bioinformatics. 2012 Dec 7;13(Suppl 17):S26. doi: 10.1186/1471-2105-13-S17-S26 (PMC3521246; doi:10.1186/1471-2105-13-S17-S26)
Supplement: Additional file 7 — Overall summary of secondary structures for ITS2 multiple alignment of Nymphaeales (Brasenia, Cabomba, Euryale, Nuphur, Nymphaea and Victoria) showing detailed information (z-score, structure conservation index, RNAz P-value, etc.) along with a Dot Plot graph. [file 1471-2105-13-S17-S26-S7.DOCX]

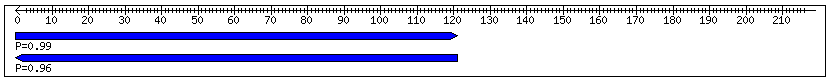

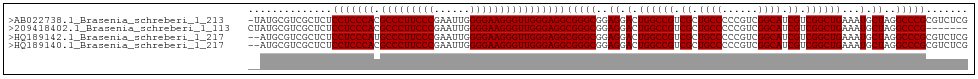

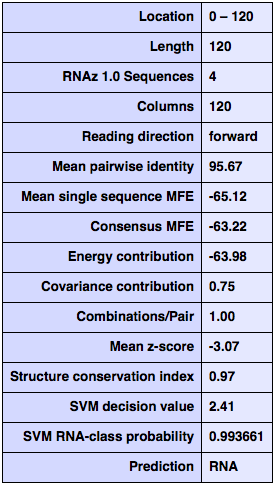

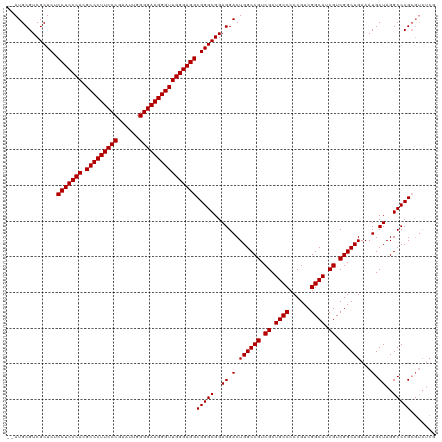

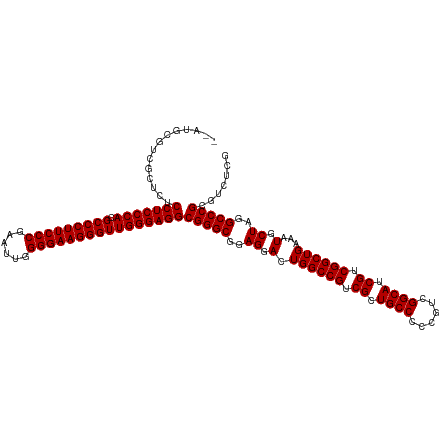


A

B


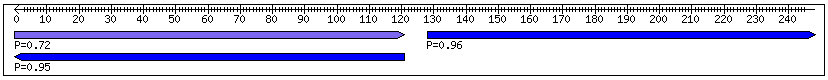

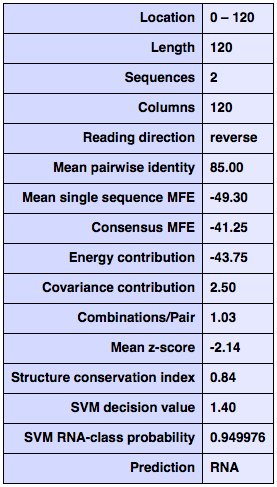


Figure S6: Overall summary of secondary structures for ITS2 multiple alignment of Nymphaeales (A. *Brasenia*, B. *Cabomba*) showing detailed information (z-score, structure conservation index, RNAz P-value, etc.) along with a Dot Plot graph


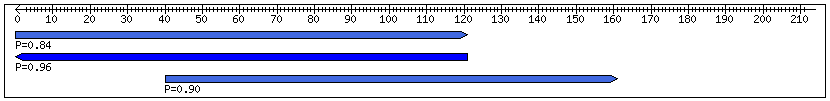

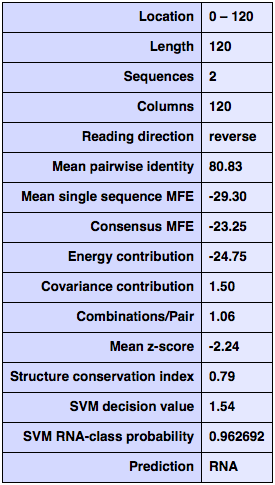

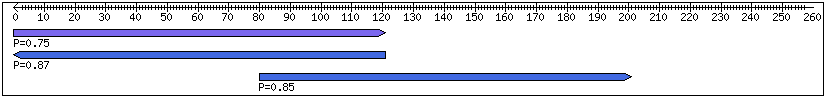

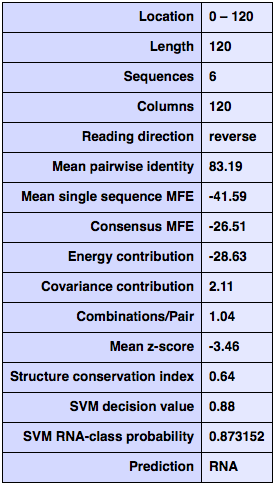


A

B

Figure S7: Overall summary of secondary structures for ITS2 multiple alignment of Nymphaeales (A. *Euryale*, B. *Nuphur*) showing detailed information (z-score, structure conservation index, RNAz P-value, etc.) along with a Dot Plot graph

A


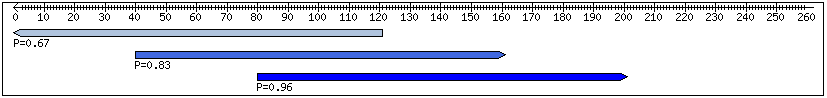

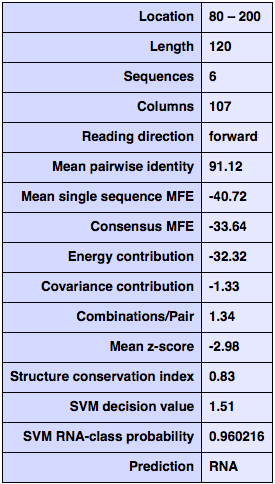


B


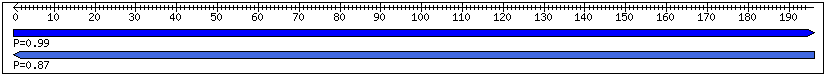

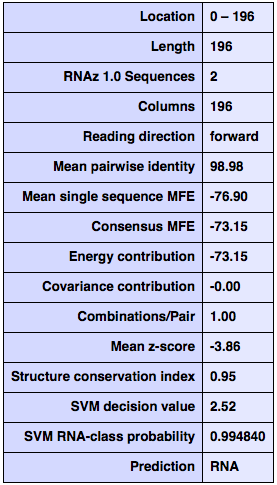


### Figure S8: Overall summary of secondary structures for ITS2 multiple alignment of Nymphaeales (A. *Nymphaea*, B. *Victoria*) showing detailed information (z-score, structure conservation index, RNAz P-value, etc.) along with a Dot Plot graph
