# Supplementary material for: Phylogenetic reconstruction in the Order Nymphaeales: ITS2 secondary structure analysis and in silico testing of maturase k (matK) as a potential marker for DNA bar coding
Source: BMC Bioinformatics. 2012 Dec 7;13(Suppl 17):S26. doi: 10.1186/1471-2105-13-S17-S26 (PMC3521246; doi:10.1186/1471-2105-13-S17-S26)
Supplement: Additional file 8 — Matlab generated mountain graph plots of Nymphaeales (ITS2 sequences). [file 1471-2105-13-S17-S26-S8.DOCX]

| 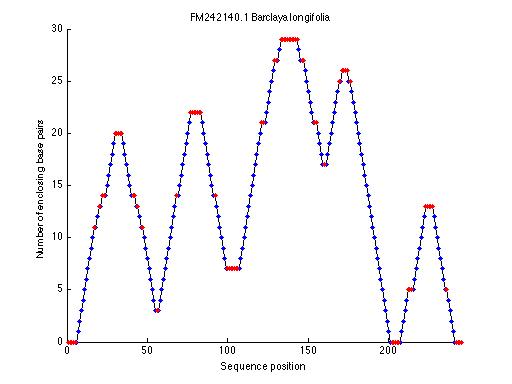 | 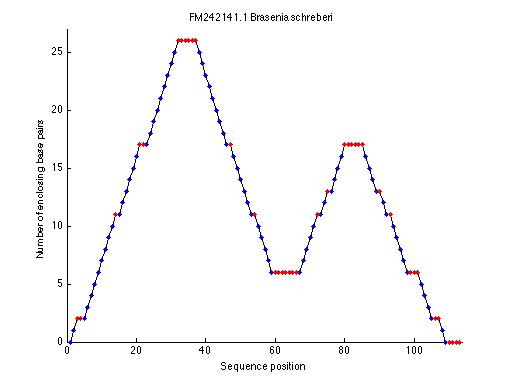 |
| --- | --- |
| 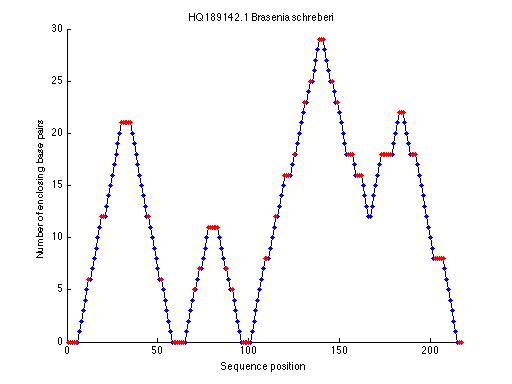 | 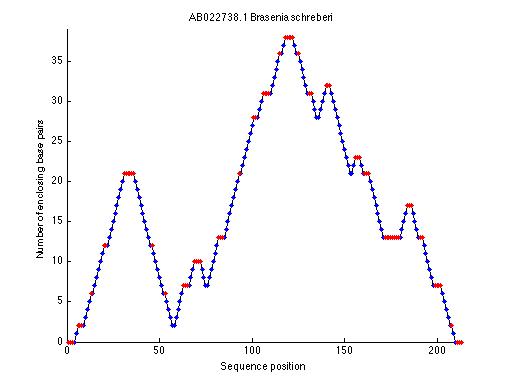 |
| 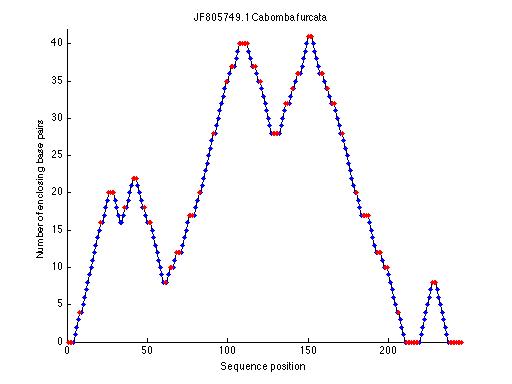 | 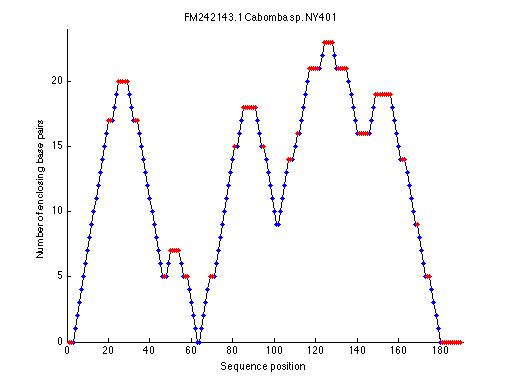 |
| 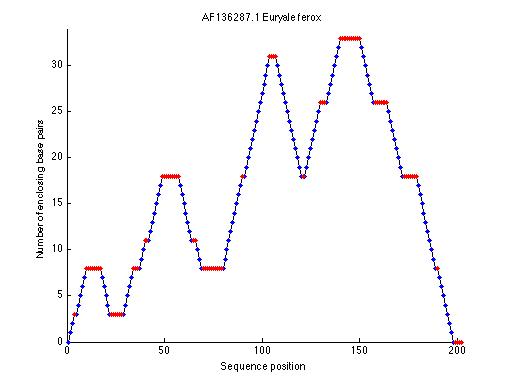 | |

Figure S9: Matlab generated mountain plot peaks of Nymphaeales (ITS2 sequences representing *Barclaya, Brasenia, Cabomba* & *Euryale* sps.)

| 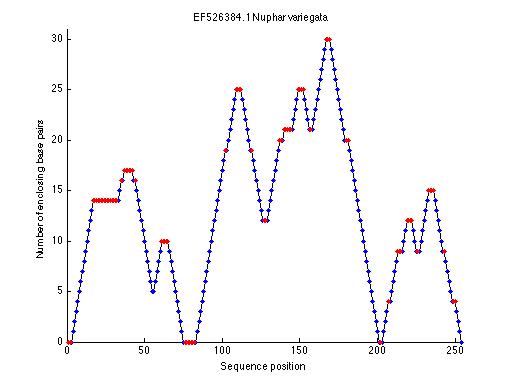 | 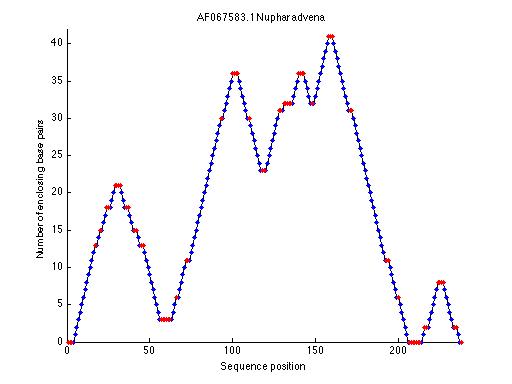 |
| --- | --- |
| 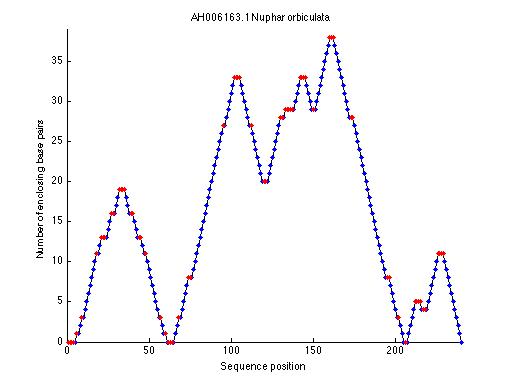 | 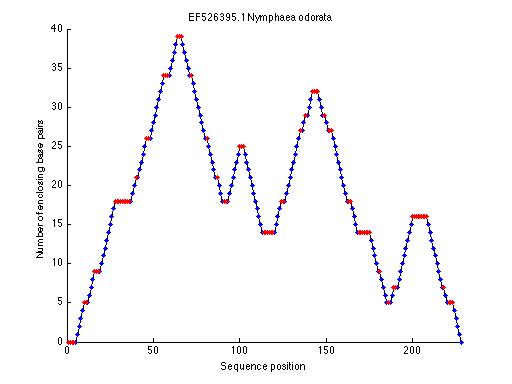 |
| 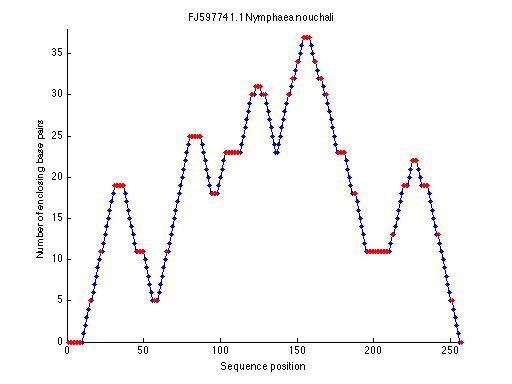 | 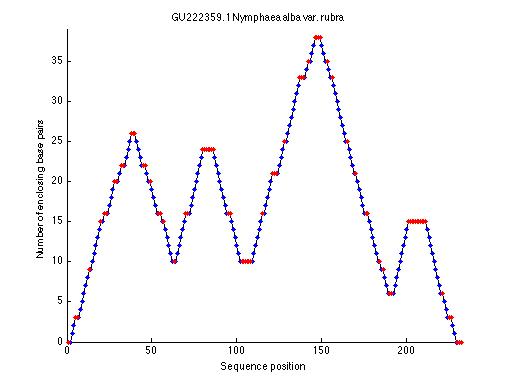 |
| 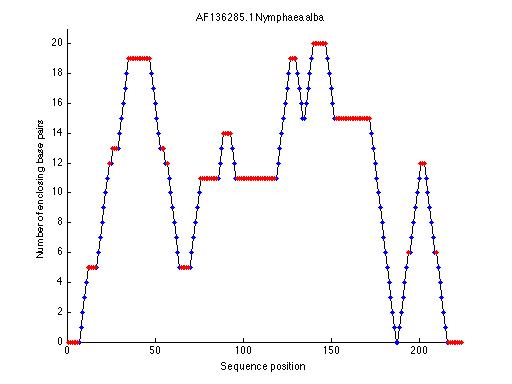 | 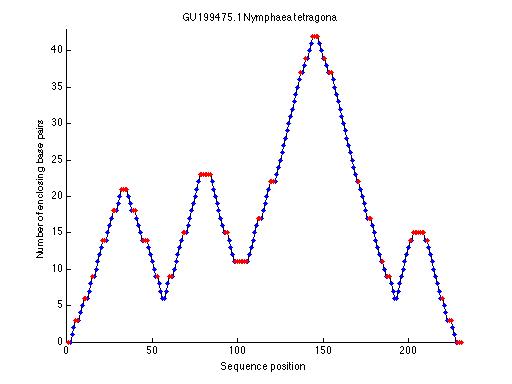 |

Figure S10: Matlab generated mountain plot peaks of Nymphaeales (ITS2 sequences representing *Nymphaea* & *Nuphar* sps.)
