# Supplementary material for: Phylogenetic reconstruction in the Order Nymphaeales: ITS2 secondary structure analysis and in silico testing of maturase k (matK) as a potential marker for DNA bar coding
Source: BMC Bioinformatics. 2012 Dec 7;13(Suppl 17):S26. doi: 10.1186/1471-2105-13-S17-S26 (PMC3521246; doi:10.1186/1471-2105-13-S17-S26)
Supplement: Additional file 9 — Nucleotide composition and GC content of matK sequences of Nymphaeales [file 1471-2105-13-S17-S26-S9.DOCX]

**Table S3: Nucleotide composition and GC content of matK sequences of Nymphaeales.**

|  | **Species** | **Accession number** | **Length** | **%GC** | **A** | **T** | **G** | **C** |
| --- | --- | --- | --- | --- | --- | --- | --- | --- |
| 1 | *Brasenia schreberi* | DQ185530.1 | 1503 | 35.9 | 457 | 506 | 258 | 282 |
| 2 | *Brasenia schreberi* | DQ185529.1 | 1502 | 35.9 | 457 | 506 | 258 | 281 |
| 3 | *Brasenia schreberi* voucher C Kim 2008 283 AJOU | HQ189139.1 | 1049 | 36.2 | 316 | 353 | 177 | 203 |
| 4 | *Cabomba caroliniana* | DQ185527.1 | 1611 | 35.3 | 496 | 546 | 270 | 299 |
| 5 | *Cabomba* sp CL 2005 | DQ185528.1 | 1606 | 35.2 | 494 | 546 | 270 | 296 |
| 6 | *Cabomba caroliniana* | AH007211.1 | 888 | 34.7 | 273 | 307 | 137 | 171 |
| 7 | *Barclaya longifolia* | DQ185534.1 | 1580 | 36.7 | 472 | 528 | 279 | 301 |
| 8 | *Barclaya longifolia* | AF092982.1 | 1516 | 36.9 | 451 | 505 | 272 | 288 |
| 9 | *Euryale ferox* | DQ185537.1 | 1523 | 35.8 | 459 | 519 | 267 | 278 |
| 10 | *Euryale ferox* | AF092994.1 | 1522 | 35.8 | 459 | 518 | 267 | 278 |
| 11 | *Trithuria austinensis* | JQ284121.1 | 935 | 35.3 | 265 | 340 | 151 | 179 |
| 12 | *Trithuria filamentosa* | JQ284137.1 | 890 | 35.6 | 252 | 321 | 141 | 176 |
| 13 | *Trithuria inconspicua* | JQ284139.1 | 890 | 35.8 | 251 | 320 | 141 | 178 |
| 14 | *Trithuria lanterna* | JQ284119.1 | 906 | 36.2 | 253 | 325 | 149 | 179 |
| 15 | *Trithuria submersa* | JQ284147.1 | 774 | 33.5 | 225 | 290 | 127 | 132 |
| 16 | *Nuphar japonica* | DQ185533.1 | 1512 | 36.7 | 452 | 505 | 268 | 287 |
| 17 | *Nuphar advena* | AY779188.1 | 1511 | 36.7 | 452 | 505 | 268 | 286 |
| 18 | *Nuphar ulvacea* | AF117068.1 | 1517 | 36.7 | 455 | 505 | 269 | 288 |
| 19 | *Nuphar orbiculata* | AH007686.1 | 933 | 35 | 291 | 315 | 149 | 178 |
| 20 | *Nuphar ozarkana* | AF117082.1 | 1517 | 36.6 | 456 | 506 | 268 | 287 |
| 21 | *Nuphar variegata* | AF092979.1 | 1517 | 36.7 | 456 | 504 | 269 | 288 |
| 22 | *Nuphar advena* | NC_008788.1 | 1518 | 36.7 | 455 | 506 | 269 | 288 |
| 23 | *Nuphar polysepala* | AF117085.1 | 1516 | 36.7 | 455 | 505 | 268 | 288 |
| 24 | *Nuphar lutea* | DQ185532.1 | 1518 | 36.6 | 455 | 507 | 268 | 288 |
| 25 | *Nuphar microphylla* | AF117094.1 | 1517 | 36.6 | 456 | 506 | 268 | 287 |
| 26 | *Nuphar pumila* | AF117088.1 | 1517 | 36.5 | 457 | 506 | 268 | 286 |
| 27 | *Nuphar oguraensis* | AF117103.1 | 1517 | 36.5 | 456 | 507 | 267 | 287 |
| 28 | *Nuphar advena* | DQ185531.1 | 1516 | 36.5 | 455 | 507 | 267 | 287 |
| 29 | *Nuphar sagittifolia* | AF117079.1 | 1451 | 430 | 489 | 254 | 278 | 36.7 |
| 30 | *Nuphar rubrodisca* | AF117097.1 | 1517 | 36.6 | 456 | 506 | 268 | 287 |
| 31 | *Nymphaea pubescens* | FJ597753.1 | 1524 | 36.5 | 454 | 513 | 271 | 286 |
| 32 | *Nymphaea rubra* | FJ597754.1 | 1524 | 36.5 | 454 | 513 | 271 | 286 |
| 33 | *Nymphaea lotus* | DQ185547.1 | 1520 | 36.1 | 456 | 515 | 267 | 282 |
| 34 | *Nymphaea lotus* voucher BioBot02117 | JQ588530.1 | 555 | 35 | 165 | 196 | 91 | 103 |
| 35 | *Nymphaea petersiana* | DQ185548.1 | 1524 | 36.3 | 454 | 517 | 267 | 286 |
|  | **Species** | **Accession number** | **Length** | **%GC** | **A** | **T** | **G** | **C** |
| 36 | *Nymphaea jamesoniana* | DQ185544.1 | 1515 | 36.2 | 456 | 511 | 264 | 284 |
| 37 | *Nymphaea amazonum* | DQ185543.1 | 1515 | 36.2 | 456 | 510 | 265 | 284 |
| 38 | *Nymphaea novogranatensis* | DQ185545.1 | 1513 | 36.2 | 455 | 510 | 265 | 283 |
| 39 | *Nymphaea oxypetala* | DQ185546.1 | 1465 | 35.9 | 438 | 501 | 252 | 274 |
| 40 | *Nymphaea tetragona* | FJ597755.1 | 1524 | 36.2 | 457 | 516 | 264 | 287 |
| 41 | *Nymphaea alba* | AJ627251.1 | 1551 | 36.1 | 466 | 525 | 271 | 289 |
| 42 | *Nymphaea odorata* | AY779190.1 | 1530 | 35.9 | 458 | 522 | 265 | 285 |
| 43 | *Nymphaea odorata* | AF092988.1 | 1529 | 36 | 458 | 521 | 265 | 285 |
| 44 | *Nymphaea odorata* | AF543742.1 | 1520 | 36.2 | 455 | 515 | 265 | 285 |
| 45 | *Nymphaea elleniae* | DQ185539.1 | 1529 | 36.4 | 457 | 515 | 268 | 289 |
| 46 | *Nymphaea caerulea* | GQ468658.1 | 1530 | 36.3 | 458 | 517 | 268 | 287 |
| 47 | *Nymphaea micrantha* | DQ185541.1 | 1529 | 36.2 | 458 | 517 | 268 | 286 |
| 48 | *Nymphaea nouchali* | GQ468659.1 | 1530 | 36.5 | 458 | 514 | 269 | 289 |
| 49 | *Nymphaea gracilis* | DQ185542.1 | 1530 | 36.4 | 458 | 515 | 269 | 288 |
| 50 | *Nymphaea macrosperma* | DQ185540.1 | 1542 | 36.1 | 463 | 522 | 267 | 290 |
| 51 | *Nymphaea odorata* | DQ185549.1 | 1530 | 35.9 | 458 | 522 | 265 | 285 |
| 52 | *Nymphaea nouchali* | FJ597752.1 | 1530 | 36.5 | 458 | 514 | 269 | 289 |
| 53 | *Nymphaea alba var. Rubra isolate NyaS1* | HQ592332.1 | 1530 | 35.9 | 458 | 522 | 265 | 285 |
| 54 | *Nymphaea pubescens isolate NypP1* | HQ592334.1 | 1524 | 36.5 | 454 | 513 | 271 | 286 |
| 55 | *Nymphaea rubra isolate NyrC1* | HQ592335.1 | 1524 | 36.5 | 454 | 513 | 271 | 286 |
| 56 | *Nymphaea x marliacea* | GQ358630.1 | 1524 | 36.1 | 457 | 517 | 266 | 284 |
| 57 | *Ondinea purpurea* | DQ185538.1 | 1522 | 36.7 | 454 | 509 | 270 | 289 |
| 58 | *Ondinea purpurea* | AH007212.1 | 1266 | 35.9 | 375 | 437 | 217 | 237 |
| 59 | *Victoria amazonica* | AF092991.1 | 1510 | 36 | 455 | 512 | 267 | 276 |
| 60 | *Victoria amazonica* x *Victoria cruziana* | DQ185536.1 | 1522 | 35.9 | 458 | 517 | 267 | 280 |
| 61 | *Victoria cruziana* | AY779189.1 | 1522 | 35.9 | 459 | 516 | 266 | 281 |
| 62 | *Cycas siamensis* | AF410165.1 | 1407 | 35.8 | 432 | 471 | 244 | 260 |
| 63 | *Cycas revoluta* | AB116583.1 | 1225 | 35.5 | 371 | 419 | 210 | 225 |
| 64 | *Ginkgo biloba* | JQ512415.1 | 1539 | 37.4 | 443 | 521 | 287 | 288 |
